# Consistent inclusion of continuum solvation in energy decomposition analysis: theory and application to molecular CO_2_ reduction catalysts†

**DOI:** 10.1039/d0sc05327a

**Published:** 2020-11-27

**Authors:** Yuezhi Mao, Matthias Loipersberger, Kareesa J. Kron, Jeffrey S. Derrick, Christopher J. Chang, Shaama Mallikarjun Sharada, Martin Head-Gordon

**Affiliations:** Department of Chemistry, University of California at Berkeley Berkeley CA 94720 USA yuezhi.mao@berkeley.edu mhg@cchem.berkeley.edu; Mork Family Department of Chemical Engineering and Material Science, University of Southern California Los Angeles CA 90089 USA; Chemical Sciences Division, Lawrence Berkeley National Laboratory Berkeley CA 94720 USA; Department of Molecular and Cell Biology, University of California Berkeley Berkeley CA 94720 USA; Department of Chemistry, University of Southern California Los Angeles CA 90089 USA

## Abstract

To facilitate computational investigation of intermolecular interactions in the solution phase, we report the development of ALMO-EDA(solv), a scheme that allows the application of continuum solvent models within the framework of energy decomposition analysis (EDA) based on absolutely localized molecular orbitals (ALMOs). In this scheme, all the quantum mechanical states involved in the variational EDA procedure are computed with the presence of solvent environment so that solvation effects are incorporated in the evaluation of *all* its energy components. After validation on several model complexes, we employ ALMO-EDA(solv) to investigate substituent effects on two classes of complexes that are related to molecular CO_2_ reduction catalysis. For [FeTPP(CO_2_-κC)]^2−^ (TPP = tetraphenylporphyrin), we reveal that two *ortho* substituents which yield most favorable CO_2_ binding, –N(CH_3_)_3_^+^ (TMA) and –OH, stabilize the complex *via* through-structure and through-space mechanisms, respectively. The coulombic interaction between the positively charged TMA group and activated CO_2_ is found to be largely attenuated by the polar solvent. Furthermore, we also provide computational support for the design strategy of utilizing bulky, flexible ligands to stabilize activated CO_2_*via* long-range Coulomb interactions, which creates biomimetic solvent-inaccessible “pockets” in that electrostatics is unscreened. For the reactant and product complexes associated with the electron transfer from the *p*-terphenyl radical anion to CO_2_, we demonstrate that the double terminal substitution of *p*-terphenyl by electron-withdrawing groups considerably strengthens the binding in the product state while moderately weakens that in the reactant state, which are both dominated by the substituent tuning of the electrostatics component. These applications illustrate that this new extension of ALMO-EDA provides a valuable means to unravel the nature of intermolecular interactions and quantify their impacts on chemical reactivity in solution.

## Abstract 2

Extension of ALMO-EDA to include contributions from continuum solvent and application to electro- and photochemical CO_2_ reduction catalysts.

## Graphical abstract

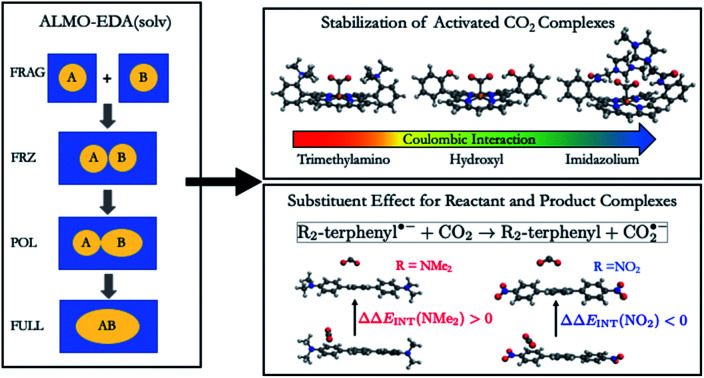

## 1 Introduction

Intermolecular interactions play an essential role in modern chemical research. Most chemical processes take place in solution, making it desirable to develop computational chemistry tools to model and analyze intermolecular interactions with solvent effects taken into account. The inclusion of solvent brings new challenges to the existing methods as solvation is able to modulate intermolecular interactions in a variety of ways. For interactions involving ionic species, the solvent helps stabilize the charged moieties while screening the long-range electrostatic interactions as a dielectric medium. Even for a neutral solute species, its electronic structure and related properties, such as multipole moments, may be altered by polar solvents, which in turn affects its interaction with other solute molecules. Such effects can impose profound influences on relative stability of intermolecular complexes as well as thermodynamics and kinetics of chemical reactions in solution.^1,2^

Implicit solvent models, which typically treat the solvent environment as a dielectric continuum and ignore its molecular-level resolution, remain widely used in modern quantum chemistry calculations to incorporate solvation effects.^3–5^ These methods are also known as self-consistent reaction field (SCRF) models, since the implicit solvent perturbs the quantum mechanical (QM) Hamiltonian *via* an external field, and the field itself depends on the QM electron density. Many variants of SCRF models that differ significantly in their formulation and complexity have been developed, including methods based on apparent surface charges (ASC),^6–16^ generalized Born models,^17–19^ and models based on direct solution of inhomogeneous Poisson–Boltzmann equations.^20–24^ The popular conductor-like (C-PCM)^8–10^ and integral-equation-formalism (IEF-PCM)^11,12^ polarizable continuum models are outstanding examples among the ASC models.

Energy decomposition analysis (EDA)^25–29^ is a powerful tool that facilitates one's understanding of intermolecular interactions by quantifying the relative importance of various physically motivated components, such as permanent electrostatics, polarization, dispersion, *etc.* While there are many perturbative or variational EDA schemes available, these developments have been focusing on intermolecular interactions in vacuum. To extend the utility of EDA approaches to intermolecular interactions under solvent environment, it is natural to integrate existing EDA schemes with implicit solvent models, considering the wide usage of the latter for describing solute–solvent interactions with minimal computational cost. The simplest approach to achieve that is to include the solvent contribution to interaction energy as an *a posteriori* correction to the gas-phase EDA result. This approach was adopted, for instance, in the EDA scheme implemented in the ONETEP^30^ linear-scaling density functional theory (DFT) program.^31,32^ While such an approach is applicable to most EDA schemes, it is not entirely satisfactory as it is unable to describe the solvation effect on each individual energy component.

In a pioneering effort to consistently incorporate solvent effect in an EDA procedure, Cammi *et al.*^33^ modified the Kituara–Morokuma (KM)-EDA^34,35^ by adding the SCRF potential of the full dimer complex to the Fock matrix that was used to generate the energy components in this EDA scheme. Similar approaches were later proposed by Contador *et al.*^36^ to study hydrogen-bonded complexes in solution, where the KM-EDA was applied to decompose interaction energies evaluated within “dimeric” cavities, and also by Gora *et al.*^37^ where the intermolecular interaction (free) energy was separated into electrostatics, exchange-repulsion, delocalization, and reaction field (solvation) contributions. Fedorov and Kitaura extended their pair-interaction (PI)-EDA scheme^38^ to treat intermolecular interactions in solution by combining the fragment molecular orbital (FMO) method^39^ with PCM models,^40^ in which they characterized two types of solvent effects: (i) screening of electrostatics and (ii) desolvation upon the formation of complex.

The EDA-PCM scheme developed by Su *et al.*,^41^ which was based upon the localized molecular orbital (LMO)-EDA scheme,^42^ is more closely related to the present work. It accounts for the solvation environment in two stages: (i) the isolated fragment orbitals (LMOs) are optimized with continuum solvent, and are then used to construct the intermediate states that are required for the evaluation of the electrostatics, exchange, repulsion, and polarization terms; (ii) a “desolvation” term, which describes the change in solute–solvent interaction energies associated with the destruction of monomer SCRFs and the formation of the full complex SCRF, is introduced in addition to the original LMO-EDA scheme. In the more recent generalized Kohn–Sham (GKS)-EDA,^43,44^ this same approach is used to incorporate the solvent contribution to the interaction free energy. While this is a rather sophisticated approach that integrates implicit solvation with a modern DFT-based EDA, the solvent reaction field is constructed only for the initial (isolated fragment) and final (full complex) states. Since it is not re-optimized for the intermediates, the solvent effect on each individual term is still not explicitly characterized.

In this paper, we integrate SCRF implicit solvent models with energy decomposition analysis of DFT calculations based on absolutely localized molecular orbitals (ALMO-EDA), whose gas-phase version was previously developed by some of us.^29,45–47^ For brevity, we denote this new extension of the ALMO-EDA for studying non-covalent interactions in solution as “ALMO-EDA(solv)” throughout this paper. The second-generation ALMO-EDA method^46,47^ partitions the total interaction energy (Δ*E*_INT_) into contributions from permanent electrostatics (ELEC), Pauli repulsion (PAULI), dispersion (DISP), polarization (POL), and charge transfer (CT):

1 Δ*E*_INT_ = Δ*E*_ELEC_ + Δ*E*_PAULI_ + Δ*E*_DISP_ + Δ*E*_POL_ + Δ*E*_CT_

where the first three terms constitute the frozen interaction energy (Δ*E*_FRZ_).^48^ This decomposition relies on the definition of two intermediate states: (i) the frozen (FRZ) state, which is constructed as an antisymmetrized product of isolated fragment wavefunctions, and (ii) the polarized (POL) state, which is obtained from variationally relaxing the frozen wavefunction with respect to orbital rotations that are “absolutely localized” on each fragment.^49,50^ Differing from the scheme previously developed by Phipps *et al.*,^32^ our new approach incorporates continuum solvent effects at all stages of the EDA procedure, namely, the isolated fragment states and FRZ, POL, and fully relaxed supersystem states. We validate and rationalize the results given by ALMO-EDA(solv) on the Na^+^⋯Cl^−^ model complex as well as the potential energy curves of two ion–water (H_2_O⋯Na^+^ and H_2_O⋯Cl^−^) complexes, in which the solvation environments are treated with C-PCM (with no empirical non-electrostatic terms)^8–10^ and the popular SMD model,^14^ respectively.

**Fig. 1:**
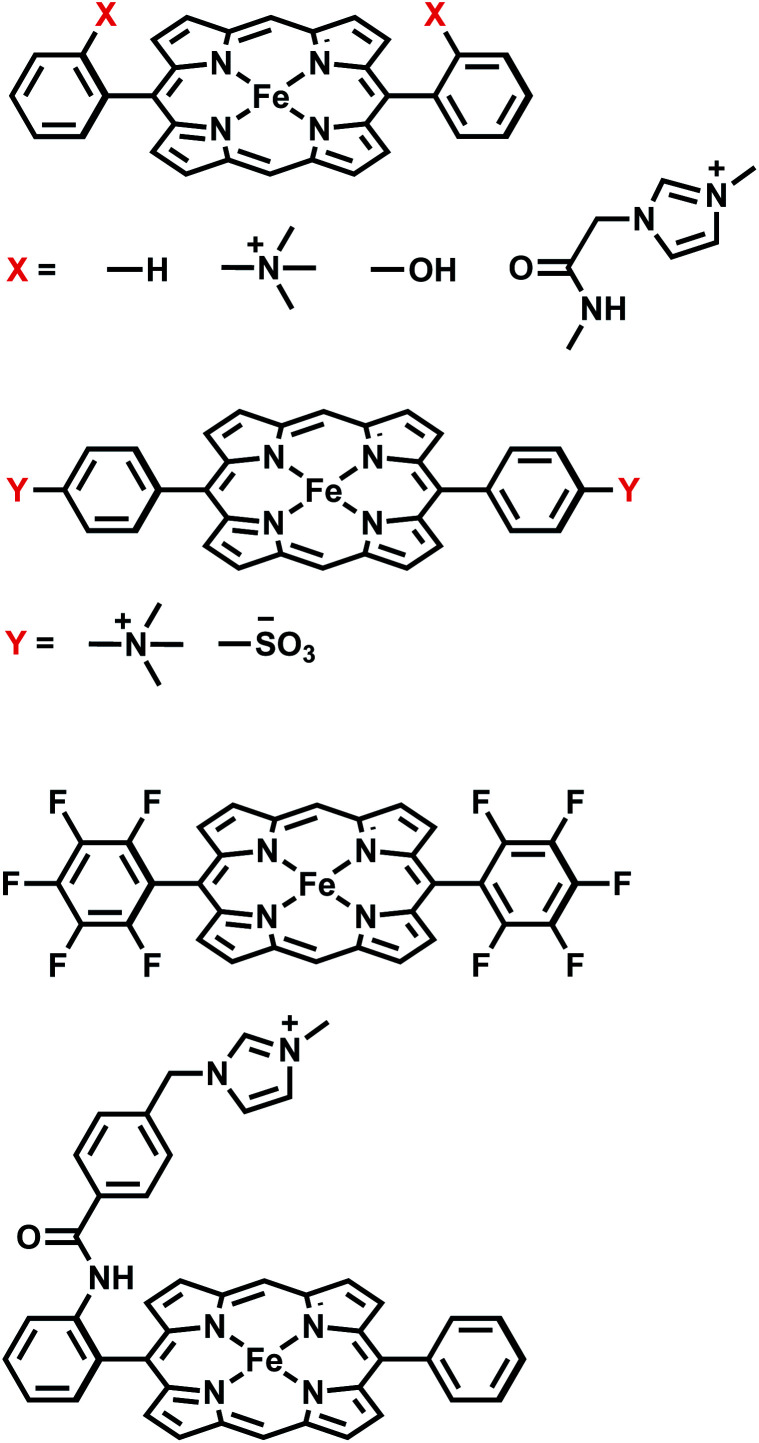
Summary of all FeTPP derivatives investigated in this study.

**Fig. 2:**
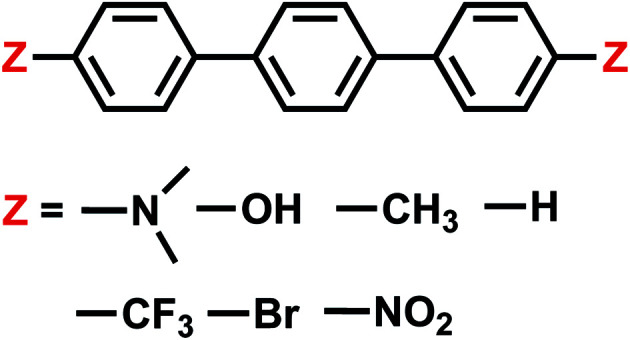
Summary of all OPP derivatives investigated in this study.

We then utilize the ALMO-EDA(solv) scheme to investigate the role of intermolecular interactions in two distinct examples of catalyzed CO_2_ reduction reactions: one is assisted by the [Fe(ii)TPP]^0^ catalyst (TPP = tetraphenylporphyrin) or its derivatives,^51^ which facilitates the 2e^−^/2H^+^ reduction of CO_2_ to CO with fast turnover rates and high product selectivity at a low overpotential^52^ by acting as an “electron mediator” between the electrode and CO_2_ in solution and stabilizing intermediates such as adducts of activated CO_2_; the other involves a single-electron transfer from a photoactivated and then reduced oligo(*p*-phenylenes) photocatalyst (OPP) to CO_2_.^53^ The catalysts investigated in this work for these two CO_2_ reduction processes are summarized in Fig. 1 and 2, respectively. Electronic structure calculations and EDA can help provide vital insights into catalytic pathways by identifying key intermediates and characterizing substrate–catalyst interactions, allowing one to understand the origin of activity or selectivity as well as the cause of any intrinsic limitation of a catalyst.^54–57^ Many CO_2_ reduction catalysts operate in aprotic polar solvents,^51–53,58^ aqueous solutions,^59^ or water/organic solvent mixtures.^60,61^ In such cases, it is essential to incorporate solvation effects in electronic structure calculations for one to obtain meaningful and reliable energetic results, especially for adducts of activated CO_2_ (CO_2_˙^−^) whose interactions with other species would be vastly different in the gas and solution phases.

## 2 Theory

**Fig. 3:**
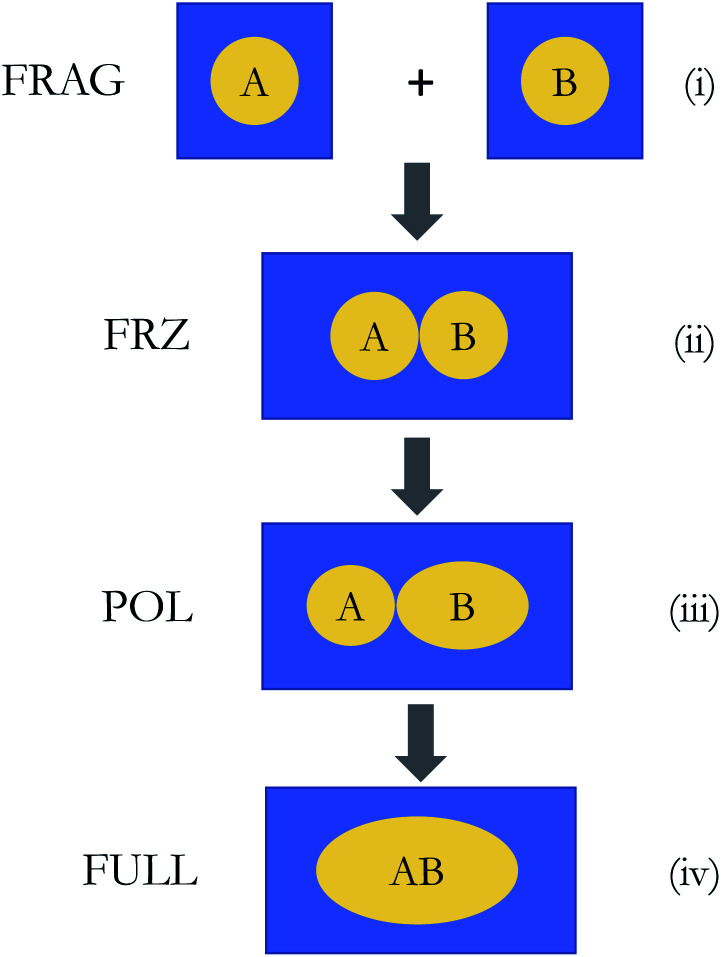
Illustration of the ALMO-EDA(solv) scheme: (i) isolated fragments that are individually solvated (the initial state); (ii) and (iii) the frozen (FRZ) and polarized (POL) intermediate states; (iv) the fully relaxed complex (the final state). Note that the shape of the molecular cavity for the complex remains the same across states (ii)–(iv), but the dielectric continuum (solvent) is polarized differently by the solute complex.

The overall procedure of ALMO-EDA(solv) is illustrated in Fig. 3, which, like the gas-phase second-generation ALMO-EDA (eqn (1)), separates the total interaction into five terms:

2 Δ*E*^(s)^_INT_ = Δ*E*^(s)^_ELEC_ + Δ*E*^(s)^_PAULI_ + Δ*E*^(s)^_DISP_ + Δ*E*^(s)^_POL_ + Δ*E*^(s)^_CT_

Here the superscript “(s)” indicates that the energetic terms are calculated with solvent taken into account. Unlike many other EDA schemes where the solvent contribution is treated as a correction to the EDA results in vacuum, our approach incorporates the solvation effect in all states (initial, intermediate and final) involved in the EDA. The interaction energy to be decomposed is given by the difference between the energy of the solvated, fully relaxed complex (stage (iv) in Fig. 3) and the sum of energies of isolated fragments that are individually solvated (stage (i) in Fig. 3), which, as in the gas-phase ALMO-EDA, can be first partitioned into contributions from frozen interaction (FRZ), polarization (POL), and charge transfer (CT):3
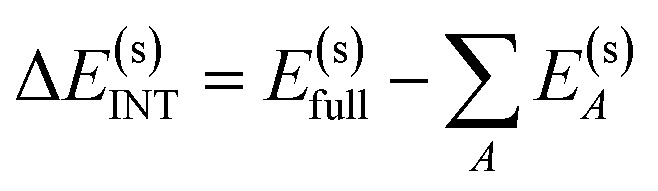

4 = Δ*E*^(s)^_FRZ_ + Δ*E*^(s)^_POL_ + Δ*E*^(s)^_CT_

The frozen interaction energy (Δ*E*^(s)^_FRZ_) describes the energy change upon the formation of a solvated complex from several individually solvated, non-interacting fragments *without* relaxing their orbitals. It corresponds to the energy change from (i) to (ii) in Fig. 1:5
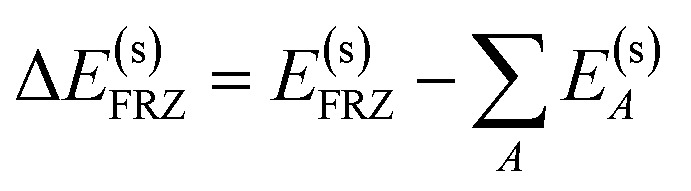
To quantify the effect of the solvent on the interaction, we introduce a new term, Δ*E*_SOL_, to describe the gain or loss of solute–solvent interaction energy upon the formation of the frozen complex:6
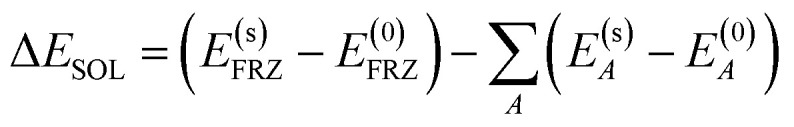
where the superscript “(0)” denotes internal electronic energies of the solute (*i.e.* excluding solute–solvent interaction, but orbitals optimized with solvent). Subtracting Δ*E*_SOL_ from Δ*E*^(s)^_FRZ_ thus recovers Δ*E*^(0)^_FRZ_, which can be further decomposed into permanent electrostatics (ELEC), Pauli repulsion (PAULI), and dispersion (DISP) contributions as in vacuum:^48^7
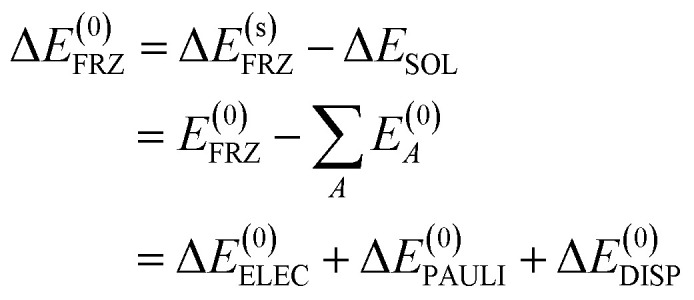


The overall decomposition of the frozen interaction energy, including solvation, is thus given by

8 Δ*E*^(s)^_FRZ_ = Δ*E*_SOL_ + Δ*E*^(0)^_ELEC_ + Δ*E*^(0)^_PAULI_ + Δ*E*^(0)^_DISP_

In this, the decomposition of the internal frozen interaction energy (Δ*E*^(0)^_FRZ_) is based on the “quasiclassical” scheme exclusively,^48,62^ where the electrostatic component, Δ*E*^(0)^_ELEC_, is defined as the Coulomb interaction between total charge distributions of isolated fragments.

For most generic implicit solvent models, the solute–solvent interaction comprises both electrostatic and non-electrostatic components. While the description of the electrostatic component plays a pivotal role in the formulation of a solvent model, the non-electrostatic solute–solvent interaction is typically described by empirical, highly parameterized functions (*e.g.* the cavity-dispersion-solvent structure (CDS) term in the SMD model^14^) and sometimes even ignored. Upon the formation of a complex, the solute–solvent electrostatic interaction may be drastically changed due to the Coulomb interaction between induced charges on different fragment cavities as well as modifications to the shape of molecular cavities. The overall effect of the change in solute–solvent electrostatic interaction, as we observe in practice, is often screening the electrostatic interaction between each fragment's “internal” charge distribution (Δ*E*^(0)^_ELEC_). The change in the non-electrostatic component of the solute–solvent interaction energy is usually of lesser importance compared to the electrostatic component, and in most cases it supplies a destabilizing effect due to the reduction of total surface area of molecular cavities upon the formation of a complex. Bearing these considerations in mind, we separate Δ*E*_SOL_ into electrostatic (Δ*E*^el^_SOL_) and non-electrostatic (Δ*E*^non-el^_SOL_) components: the former is combined with Δ*E*^(0)^_ELEC_, giving rise to a solvent-corrected electrostatic term that is denoted as Δ*E*^(s)^_ELEC_, and the latter is combined with Δ*E*^(0)^_PAULI_ because of their common short-ranged nature. The decomposition of the frozen term in ALMO-EDA(solv) (eqn (8)) can thus be rewritten as9
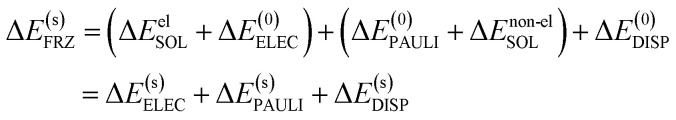


One should note that in eqn (9) we have assumed that Δ*E*^(s)^_DISP_ ≈ Δ*E*^(0)^_DISP_, that is, the dispersion interaction between fragments is unaffected by the presence of solvent except that the fragment wavefunctions are optimized with solvent. This assumption is plausible when the two interacting moieties are in close contact, especially when they reside in the same molecular cavity, but may become less justified when the two moieties are well-separated and reside in two non-overlapping cavities, since dispersion interaction, which can be viewed as interactions between fluctuating dipoles, may also be screened by the dielectric medium.^63^ This many-body dispersion effect^64^ seems non-trivial to include in a continuum solvent model, so we stick with this assumption for now and decompose the frozen interaction energy based on eqn (9) in the rest of this paper.

The polarization energy (Δ*E*^(s)^_POL_) in ALMO-EDA(solv) is defined as the energy difference between the polarized intermediate state and the solvated frozen complex, which corresponds to the energy change from stage (ii) to (iii) in Fig. 3:

10 Δ*E*^(s)^_POL_ = *E*^(s)^_POL_ − *E*^(s)^_FRZ_

It describes the energetic stabilization associated with the intramolecular relaxation of each fragment in the presence of other fragments as well as the solvent environment. In DFT-based ALMO-EDA, the POL state is obtained by variationally minimizing the supersystem energy subject to the constraint that the polarized molecular orbitals (MOs) of each fragment are expanded in fragment-specific basis functions (either atomic orbitals (AO) or frozen occupied MOs plus fragment electrical response functions^50^), which is known as the “self-consistent field for molecular interaction” (SCF-MI) approach.^49,65,66^ In each iteration of the SCF-MI calculation, the solvent reaction field will be re-equilibrated in accord with the updated electron density of the complex, ensuring that the response of the solvent to the change in solute electronic structure is incorporated in a self-consistent manner.

Finally, we perform a standard, unconstrained SCF calculation within the solvent environment, and the energy lowering relative to the POL state (from (iii) to (iv) in Fig. 3) is defined as the charge-transfer (CT) energy:

11 Δ*E*^(s)^_CT_ = *E*^(s)^_full_ − *E*^(s)^_POL_

Note that the charge transfer process might be associated with charge redistribution in the complex, which in turn induces responses in the reaction field. Such an effect is captured by the self-consistent minimization of *E*^(s)^_full_ in the presence of solvent.

In summary, the energy decomposition given by the ALMO-EDA(solv) scheme is formally identical to its gas-phase counterpart. The explicit change in solute–solvent interaction energy upon the formation of complex is reflected in Δ*E*_SOL_, which can be further partitioned into electrostatic and non-electrostatic components that serve as corrections to the internal ELEC and PAULI terms, respectively. The POL and CT contributions to the interaction are calculated with all involved intermediate states properly solvated, and hence the solvent effect on these terms will also be taken into account.

## 3 Computational details

We implemented the ALMO-EDA(solv) scheme in a locally modified Q-Chem 5.2 software package,^67^ which serves as an extension of the original routines for the second-generation ALMO-EDA for DFT calculations.^46,47^ While this scheme, in principle, should be compatible with most of the available SCRF models, in this work we demonstrate it with two widely used approaches: the conductor-like PCM (C-PCM)^8–10^ and the SMD model.^14^ The non-electrostatic effects of the solvent were ignored in our calculations using C-PCM, whereas the solvation free energy (Δ*G*_S_) produced by SMD comprises both electrostatic and non-electrostatic contributions, which correspond to the “electronic-nuclear-polarization” (ENP) and the “cavity-dispersion-solvent structure” (CDS) terms, respectively:^14^Δ*G*_S_ = Δ*G*_ENP_ + Δ*G*_CDS_In the Q-Chem implementation of the SMD model, IEF-PCM^11,12^ is employed to describe the solute–solvent electrostatic interaction.

Both C-PCM and IEF-PCM require solving for discretized point charges on the surface of a molecular cavity. In our calculations using C-PCM, the molecular cavities were constructed using the union of a series of atom-centered spheres whose radii are determined using each atom's van der Waals radius from the Universal Force Field^68^ scaled by a factor of 1.2. The boundary between solute and solvent constructed thereof is known as the solvent accessible surface (SAS). The calculations using SMD construct the molecular cavities in a similar manner but use its own set of atomic radii.^69^ To ensure the smoothness of potential energy surfaces (PESs) associated with molecular complexes, the switching/Gaussian method developed by Lange and Herbert^15,16^ was employed in both C-PCM and SMD calculations, in which the atomic spheres are discretized using Lebedev grids rather than more traditional tessellation schemes (*e.g.* the GEPOL algorithm^70^). In this work, 302 Lebedev points were used for all atoms in our calculations using C-PCM or SMD.

Unless otherwise specified, the second-generation ALMO-EDA calculations are performed with the *ω*B97X-V functional,^71^ which was shown to be one of the best-performing functionals for non-covalent interactions *via* extensive benchmarks^72–74^ and gave excellent results in our previous studies involving systems such as ion–water complexes.^62,75,76^ The DFT calculations employ a (99, 590) grid (99 radial shells with 590 Lebedev points on each) for the integration of the exchange–correlation (XC) functional and the SG-1 grid^77^ for the VV10 non-local correlation functional^78^ in *ω*B97X-V. The decomposition of the frozen interaction energy in ALMO-EDA calculations follows the “quasiclassical” scheme^48,62^ exclusively in this work, as we have noted in Section 2. For the separation between POL and CT, the more sophisticated fragment electrical response function (FERF) method^50^ featuring a well-defined basis set limit was used for the small model systems (Sections 4.1 and 4.2), while the original ALMO scheme based on partition of the AOs^45,49^ was used for the applications in Sections 4.3 and 4.4 with more moderate basis sets (def2-TZVPP and def2-TZVPD,^79,80^ respectively) given the substantial sizes of these systems.

## 4 Results

### 4.1 The sodium-chloride model complex

To validate the treatment of solvent effects in ALMO-EDA(solv), we first investigate a Na^+^⋯Cl^−^ model complex where the two ions are separated by 20 Å and immersed in solvent with varying dielectric constant described by C-PCM. At the asymptotic limit, the strength of the electrostatic interaction between Na^+^ and Cl^−^ in a dielectric medium is 1/*ε* of that in vacuum according to Coulomb's law, where *ε* is the (relative) dielectric constant of the medium. This relation is reproduced by ALMO-EDA(solv) as demonstrated in Table 1. The Δ*E*^(0)^_ELEC_ term reflects the strength of the Coulomb interaction in vacuum, while Δ*E*^el^_SOL_ is the correction from solute–solvent electrostatic interaction, which is an unfavorable term as its net effect is to damp the attractive Coulomb interaction between Na^+^ and Cl^−^. The sum of these two terms obtained in EDA calculations gives the effective (screened) electrostatic interaction in solution, Δ*E*^(s)^_ELEC_, whose relative strength against Δ*E*^(0)^_ELEC_ shows excellent agreement with the dielectric constants (*ε*) specified as an initial parameter in these calculations. This agreement can also be reproduced with the widely used IEF-PCM approach (see Table S1 in the ESI†), confirming that the treatment of solvent-screened permanent electrostatics in our EDA shows correct asymptotic behavior.

**Table 1:** Energetic contributions (in kJ mol^−1^) from internal QM electrostatics (Δ*E*^(0)^_ELEC_) and solute–solvent electrostatic interaction (Δ*E*^el^_SOL_) to the interaction between Na^+^ and Cl^−^ which are separated by 20 Å from each other. The calculations are performed using *ω*B97X-V/def2-TZVPPD and C-PCM with varying dielectric constants. Δ*E*^(s)^_ELEC_ = Δ*E*^(0)^_ELEC_ + Δ*E*^el^_SOL_ is the effective (screened) electrostatic interaction in solution

*ε*	Δ*E*^(0)^_ELEC_	Δ*E*^el^_SOL_	Δ*E*^(s)^_ELEC_	Δ*E*^(0)^_ELEC_/Δ*E*^(s)^_ELEC_
1	−69.47	0.00	−69.47	1.0
10	−69.47	62.52	−6.95	10.0
20	−69.47	65.99	−3.48	20.0
40	−69.47	67.73	−1.74	40.0
80	−69.47	68.60	−0.87	79.9

**Fig. 4:**
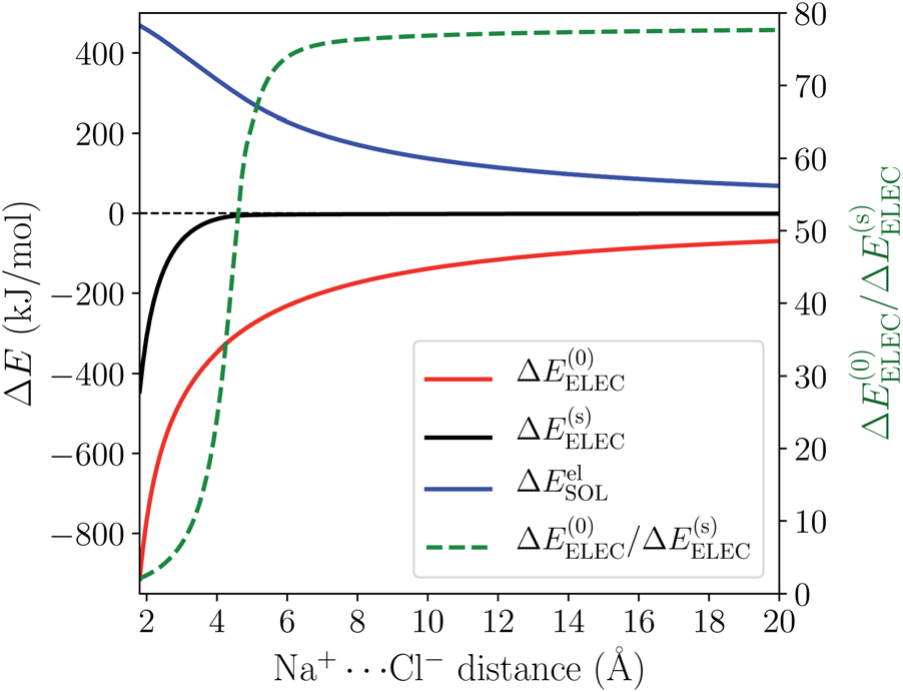
Left *y*-axis: distance dependence of Δ*E*^(0)^_ELEC_, Δ*E*^el^_SOL_, and Δ*E*^(s)^_ELEC_ for the Na^+^⋯Cl^−^ model complex in C-PCM water in the range of 1.8–20.0 Å; right *y*-axis: variation of the Δ*E*^(0)^_ELEC_/Δ*E*^(s)^_ELEC_ ratio with the Na^+^⋯Cl^−^ distance (plotted with the green dashed curve).

The distance dependence of the three electrostatics-related terms in Table 1 is shown in Fig. 4, where H_2_O (*ε* = 78.2) described by C-PCM is employed as the implicit solvent. At long range, the attraction between Na^+^ and Cl^−^ is subjected to strong solvent screening, which renders the effective electrostatic interaction in solution minimal (*e.g.* at 5 Å Δ*E*^(s)^_ELEC_ is only −4.3 kJ mol^−1^). Furthermore, the Δ*E*^(0)^_ELEC_/Δ*E*^(s)^_ELEC_ ratio stays close to the asymptotic limit (78.2) in this range, albeit with more pronounced deviation when moving closer. In contrast, at shorter distances (<5 Å) this ratio decreases rapidly indicating less effective screening of the attractive electrostatics. At the minimum-energy distance (2.5 Å), Δ*E*^(s)^_ELEC_ gains appreciable magnitude (−140 kJ mol^−1^) and the value of Δ*E*^(0)^_ELEC_/Δ*E*^(s)^_ELEC_ lowers to 4.1. Further examining the distance dependence of Δ*E*^el^_SOL_ reveals an inflection point at 3.35 Å, *i.e.*, the curve starts to *flatten* when moving to shorter distances.

**Fig. 5:**
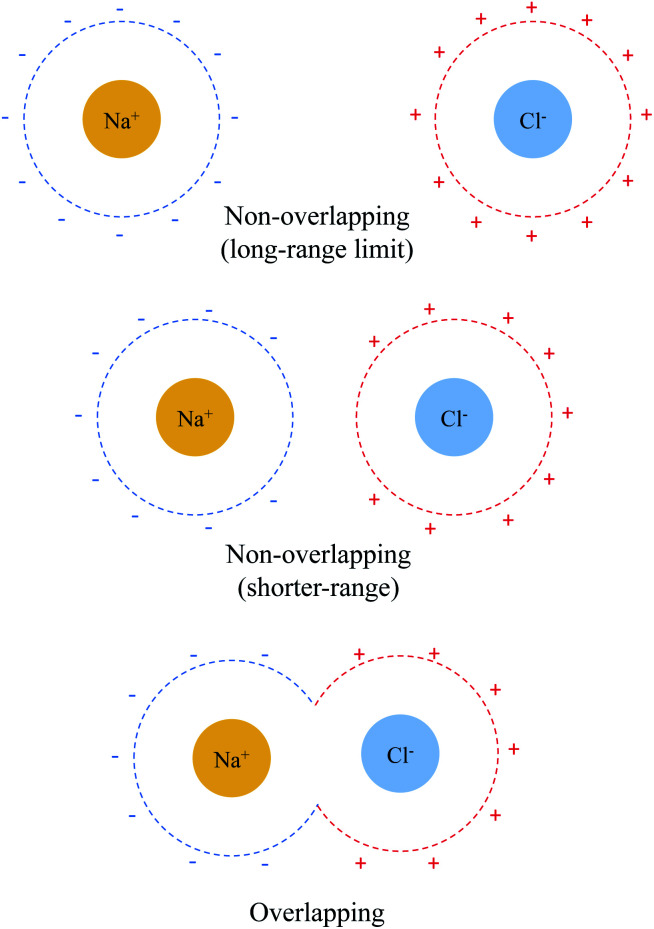
Schematic illustration of solvent effect on the Na^+^⋯Cl^−^ electrostatic interaction in the non-overlapping (upper and middle panels) and overlapping (lower panel) regimes. The dashed lines depict the cavities for each solute and “+” and “−” represent positive and negative surface charges, respectively.

The sharp contrast between the short- and long-range behavior of these terms can be rationalized schematically with Fig. 5. In the long-range limit (upper panel), the cavities for Na^+^ and Cl^−^ are well-separated and the surface charges are mainly induced by their respective nuclei and electrons, yielding strong solvent screening of the long-range electrostatics. With the decrease in inter-fragment distance, even before the cavities start to overlap, the surface charges on each cavity will be influenced by both solutes (Na^+^ and Cl^−^) simultaneously, which then largely cancel each other in the inter-fragment region (see mid-panel of Fig. 5). This would result in weakened screening and potentially explains the modest increase in the deviation from the long-range limit for Δ*E*^(0)^_ELEC_/Δ*E*^(s)^_ELEC_. Finally, when the two cavities start to overlap and merge with each other (for this system it occurs at 4.8 Å with our computational setup for PCM), as shown in the lower panel of Fig. 5, the dielectric solvent between the two fragments is “squeezed out” and the screening becomes even more incomplete. In addition, the inter-penetration of QM electron densities in the overlapping regime enhances the internal electrostatic attraction between Na^+^ and Cl^−^, which further contributes to the decrease in the value of Δ*E*^(0)^_ELEC_/Δ*E*^(s)^_ELEC_ since this short-ranged effect is subjected to almost no solvent screening. These effects, all together, result in only partial screening of the attractive internal electrostatics as well as the inflection point in the magnitude of Δ*E*^el^_SOL_ at short Na^+^⋯Cl^−^ distances.

### 4.2 Potential energy curves for ion–water interactions

As a further validation of ALMO-EDA(solv), we employ it to investigate the distance dependence of ion–water interactions (H_2_O⋯Na^+^ and H_2_O⋯Cl^−^) in three different solvents: toluene (*ε* = 2.37), acetonitrile (*ε* = 37.5), and water (*ε* = 78.3). The gas-phase ALMO-EDA results for these systems are available in ref. 62. The long-range electrostatics in these systems can be depicted as charge-dipole interactions (*R*^−2^), and in the short range polarization also contributes significantly to binding (especially in the H_2_O⋯Na^+^ case). These strong interactions, however, will be diminished in solution due to solvent screening.

**Fig. 6:**
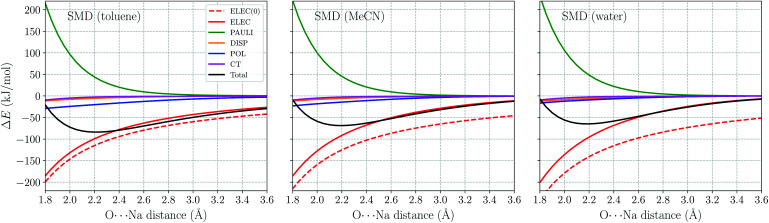
ALMO-EDA(solv) results (in kJ mol^−1^) for the H_2_O⋯Na^+^ complex in toluene, acetonitrile (MeCN), and water solutions with the O⋯Na^+^ distance ranging from 1.8 to 3.6 Å. All calculations are performed using *ω*B97X-V/def2-TZVPPD with the SMD solvent model. Terms in ALMO-EDA(solv) are represented with solid lines while the internal electrostatic interaction, denoted as “ELEC(0)”, is shown as a dashed line.

Fig. 6 shows the *ω*B97X-V/def2-TZVPPD total interaction energy and its components for the H_2_O⋯Na^+^ complex *vs. r*_O–Na_ in the range of 1.8–3.6 Å. Permanent electrostatics makes the largest contribution to binding at this full range despite solvent screening. Comparing the results for different solvents, one remarkable feature is that the internal electrostatic interaction, ELEC(0), becomes more favorable with the increase in solvent dielectric constant (from left to right in Fig. 6). This is because the dipole moment of an isolated H_2_O molecule increases when placed in a more polar solvent environment. Nonetheless, since more polar solvent also screens more strongly, the total electrostatic interaction (ELEC) shows similar strength in both solvents around equilibrium (∼2.2 Å). The strength of long-range electrostatics and the total interaction energy, on the other hand, is governed by solvent screening, as evidenced by the smaller magnitude of ELEC at 3.6 Å in water *vs.* toluene in Fig. 6.

Another energy component that is strongly impacted by solvent effects is polarization (POL). According to ref. 62, the gas-phase polarization energy for H_2_O⋯Na^+^ at 2.2 Å is around −25 kJ mol^−1^, while this value reduces to around −9 kJ mol^−1^ in SMD water. Similar to long-range electrostatics, POL becomes less diminished when the solvent is less polar. The other three energy components, PAULI, DISP, and CT show much smaller variance with the change of solvent. They are apparently less affected by the solvent properties, at least within the present ALMO-EDA(solv) model.

Within the SMD model, the solvent contribution to binding can be further partitioned into electrostatic and non-electrostatic contributions. The distance dependence of these two terms as well as that of the overall Δ*E*_SOL_ is shown in Fig. S1 in the ESI.† The non-electrostatic (CDS) term has minimal significance compared to the electrostatic contribution and vanishes in the long range when the cavities of two fragments are fully separated. It is noteworthy that the non-electronic contribution to binding does *not* have a definite sign: among the three solvents here, Δ*E*^non-el^_SOL_ is exclusively positive for toluene while negative for acetonitrile and water. This is presumably a consequence of the CDS term in SMD aiming to account *in aggregate* for non-electrostatic solvation effects to better reproduce experimental solvation free energies.

The solvent electrostatic contribution (Δ*E*^el^_SOL_), on the other hand, is repulsive over the full range (1.8–8.0 Å) for all three solvents. As in the Na^+^ ⋯Cl^−^ case (Fig. 4(a)), this contribution first increases rapidly with the shortening of intermolecular distance, which damps the increasingly attractive internal electrostatic interaction. Δ*E*^el^_SOL_ is more repulsive in solvents with larger dielectric constants, indicating their stronger screening capability. Moving into the overlapping regime, the solvent electrostatic term flattens first and then reaches a maximum when the O⋯Na^+^ distance is slightly below 3 Å, *i.e.*, the magnitude of Δ*E*^el^_SOL_ starts to decrease when the intermolecular distance is further shortened. This maximum in the solvent electrostatic contribution was also revealed by Su *et al.* with their LMO-EDA-PCM scheme for the water dimer.^41^ We attribute this behavior to the merging of fragment cavities upon the formation of complex, which leads to diminished screening of the internal electrostatic interaction (*vide supra*).

In the ESI† we also show the analogues of Fig. 6 and S1† for the H_2_O⋯Cl^−^ complex (Fig. S2 and S3†). The ELEC and POL terms obtained from ALMO-EDA(solv) with different solvents (Fig. S2†) show similar trends as in the H_2_O⋯Na^+^ case except that the relative strength of solvent screening (indicated by their dielectric constants) has a more substantial impact on the electrostatic interaction and total interaction energy around the equilibrium distance, rendering the intermolecular binding notably stronger in the least polar solvent (toluene). In addition, the location of the maximum in the solvent electrostatic contribution (Δ*E*^el^_SOL_) varies from solvent to solvent: it appears at a notably longer O⋯Cl distance in H_2_O than in the other two solvents. This distinction between solvents is more pronounced than in the H_2_O⋯Na^+^ case where the maxima in Δ*E*^el^_SOL_ with different solvents appear at similar distances.

### 4.3 Substituent effects on the stability of [FeTPP(CO_2_˙^−^)] complexes: through-space *vs.* through-structure mechanisms

There is tremendous research interest in homogeneous electrochemical reduction of CO_2_,^81–83^ because CO_2_ conversion to carbon-based fuels could underpin a future carbon-neutral economy. The initial step is activating CO_2_, whose one-electron reduction potential is quite unfavorable (−1.90 V *vs.* NHE) by comparison with the reduction potential to convert CO_2_ to more reduced products such as the 2e^−^ reduction to CO (−0.53 V *vs.* NHE). The first function of a catalyst is thus to stabilize the activated CO_2_ as it drives the first reduction, and thereby reduce the thermodynamic overpotential. Molecular catalysts are of great interest for this purpose, in addition to enhancing turnover rates, and suppressing competitive side reactions such as the hydrogen evolution reaction (HER). Among available transition metal based catalysts, iron complexes have received particular attention because of the earth abundance of Fe and their low toxicity. To date, several iron catalysts with different ligand frameworks have been developed for the reduction of CO_2_.^57,60,84–86^ The most prominent family is [Fe(ii)TPP]^0^ (TPP = tetraphenylporphyrin)^87,88^ and its derivatives.^51,89–92^ Mechanistic studies indicate that stabilizing the activated CO_2_ adduct intermediate can substantially improve the performance of the catalyst.^51,58,93^ An “electronic scaling relationship” (so-called because it reflects electronic substituent effects) between overpotential and turnover frequency (TOF) was previously established by stepwise fluorination of the phenyl groups in the TPP ligand framework.^90^ The four derivatives FeTPP, FeF5TPP, FeF10TPP and FeF20TPP (full fluorination of zero, one, two, and four phenyl rings, respectively) show a linear correlation between a favorable decrease in overpotential and an unfavorable decrease in TOF. This effect stems from stronger inductive effects that accompany the addition of –F substituents, which was referred to as a “through-structure” electronic effect.^90^

A subsequent experimental study^51^ further demonstrated that charged substituents can break electronic scaling relationships. Introducing a positively charged trimethylammonio (TMA, –NMe_3_^+^) group to either the *ortho* or *para* position of each phenyl group yields tetra-trimethylanilinium–porphyrin complexes (Fe-*o*-TMA or Fe-*p*-TMA). Unlike the fluorinated complexes, Fe-*o*-TMA exhibits high TOFs at low overpotentials. In contrast, introducing the negatively charged sulfonate (–SO_3_^−^) group to the *para* positions to yield Fe-*p*-SUL results in lower TOF at a higher overpotential relative to FeTPP and Fe-*o*/*p*-TMA. Based on these results, the authors hypothesized that in these cases the strength of CO_2_ binding is not only controlled by the through-structure inductive effect of the electron-withdrawing groups but more importantly modulated by the long-range (through-space) electrostatic interactions between these charged substituents and the negatively charged CO_2_˙^−^ moiety. This suggested mechanism could explain why Fe-*o*-TMA catalyzes CO_2_ reduction at such high TOFs.^51^ Further evidence for the importance of stabilizing the activated CO_2_ adduct is provided by the *ortho* hydroxyl substituted TPP complex, tetrakis-(2′,6′-dihydroxyphenyl)-porphyrin (CAT).^89^ This derivative is also a more active catalyst than unsubstituted FeTPP, where the stabilization of activated CO_2_ may also result from favorable (through-space) interactions between the hydroxyl groups and negatively charged CO_2_˙^−^.

**Fig. 7:**
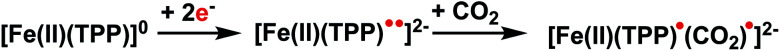
Simplified steps in the catalytic pathway of FeTPP leading to the activated CO_2_ intermediate.

**Fig. 8:**
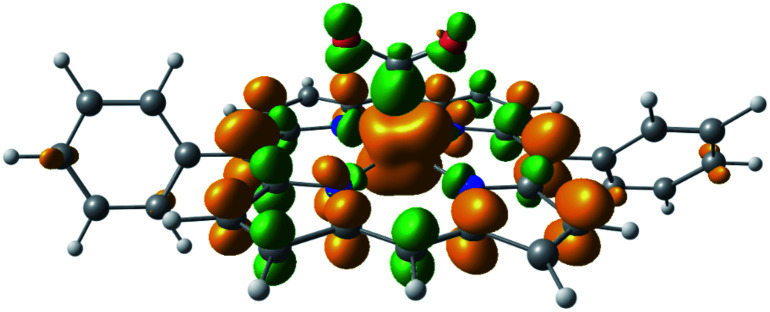
Spin density of the doubly reduced CO_2_ adduct [FeTPP(CO_2_-κC)]^2−^ (green: excess *α* spin; gold: excess *β* spin).

Here we employed the ALMO-EDA(solv) approach to gain insights into the stabilization mechanisms of activated CO_2_ in the [FeTPP(CO_2_-κC)]^2−^ adducts arising from the reaction of CO_2_ with the doubly reduced FeTPP ([FeTPP]^2−^, which is formally Fe(0)) complex (see Fig. 7 for a simplified reaction scheme). The solvent MeCN was described by C-PCM with the dielectric constant set to 35.88.^94^ The electronic ground state of the complex is a (broken-symmetry) triplet in an η^1^-κC binding mode, whose spin density contours (Fig. 8) reveal a significant amount of excess spin in the ligand framework as well as on the CO_2_ moiety indicating reduction of both the CO_2_ and the non-innocent TPP ligand. These reduced moieties are both antiferromagnetically coupled to the metal center as previously discussed in ref. 58. Therefore, this complex can be best represented as [Fe(ii)(TPP˙^3−^)(CO_2_˙^−^-κC)]^2−^. For brevity, in the following discussion we omit the “κC” notation that specifies the binding mode of CO_2_ to the metal center.

**Fig. 9:**
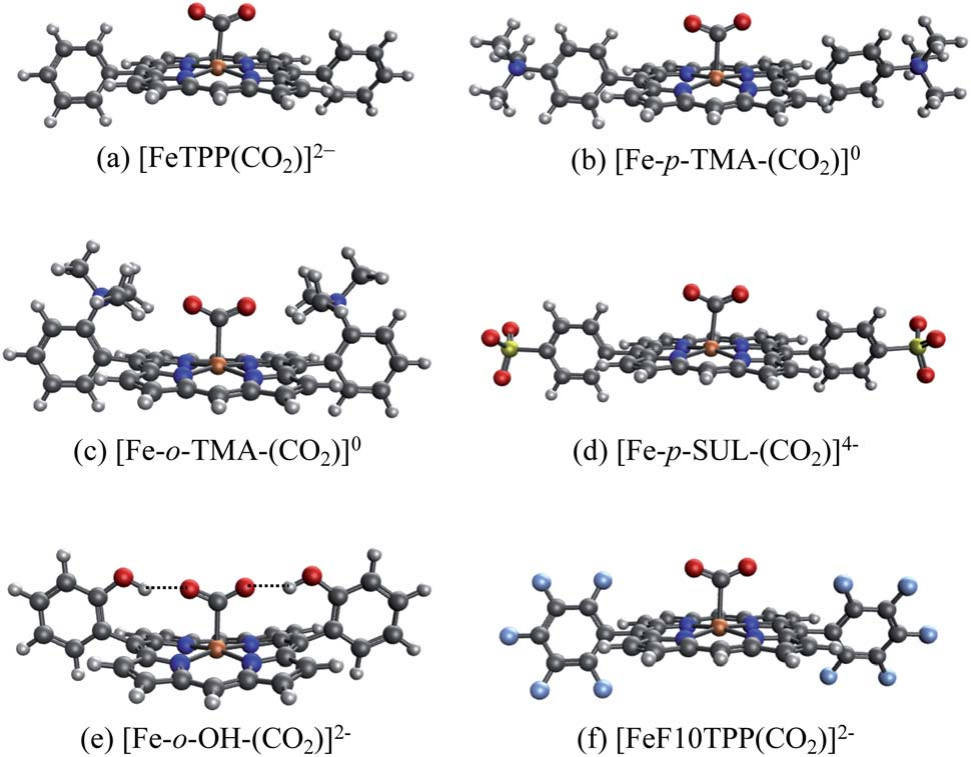
Optimized geometries for the [FeTPP(CO_2_-κC)]^2−^ adduct with varying substitutions: (a) unsubstituted (–H); (b) *para*-trimethylammonio (–NMe_3_^+^); (c) *ortho*-trimethylammonio (–NMe_3_^+^); (d) *para*-sulfonate (–SO_3_^−^); (e) *ortho*-hydroxyl (–OH); (f) all-fluorinated (–F).

We illustrate the effects of different substituents by comparing the EDA results of complexes with varying substitutions (–H, –NMe_3_^+^, –SO_3_^−^, –OH) on the phenyl rings. To reduce the computational expense, we truncated these systems by removing two of the four phenyl groups based on the fact that the CO_2_ moiety is positioned in such a way that only two substituent groups can strongly interact with it (see Fig. 9). In total, we compare six different CO_2_ adducts with varying net charges due to the distinction in substituents: (a) unsubstituted ([FeTPP(CO_2_)]^2−^), (b) *para* –NMe_3_^+^ substituted ([Fe-*p*-TMA-(CO_2_)]^0^), (c) *ortho* –NMe_3_^+^ substituted ([Fe-*o*-TMA-(CO_2_)]^0^), (d) *para* –SO_3_^−^ substituted ([Fe-*p*-SUL-(CO_2_)]^4−^), (e) *ortho* –OH substituted ([Fe-*o*-OH-(CO_2_)]^2−^), and (f) perfluorinated ([FeF10TPP(CO_2_)]^2−^). The geometries of these complexes are shown in Fig. 9.

The results of the EDA calculations depend on the choice of reference states of both fragments, *i.e.*, the electronic states they are in when they are infinitely separated. In this example two fragmentation schemes are plausible: (i) the “natural” fragmentation that corresponds to reactants in the catalytic cycle, where a neutral CO_2_ (*S* = 0) molecule is bound to a doubly reduced iron complex (*S* = 1); (ii) fragmentation based on the charge population of the final product complex, giving a singly reduced metal complex (*S* = 3/2) and a singly reduced (activated) CO_2_ radical anion (CO_2_˙^−^, *S* = 1/2). Our results show that the former fragmentation scheme is unsuitable here due to the drastic geometry distortion energy (>200 kJ mol^−1^) that is associated with the bending of a neutral CO_2_ molecule, which would lead to EDA results that are dominated by this geometry distortion term and are thus less insightful (see Table S2 in the ESI†). In contrast, the geometry distortion term associated with the CO_2_˙^−^ moiety in the latter fragmentation scheme is minimal (less than 1 kJ mol^−1^). Therefore, we selected the second fragmentation scheme in the following discussion, which corresponds to the binding of CO_2_˙^−^ with singly reduced FeTPP and its substituted derivatives.

Our choice yields (up to) two charged fragments where the net charge on the FeTPP moiety depends on the substituents. A comparison across all these compounds without considering the solvent effect would lead to unreasonably large variations in total interaction energy due to the large variation in gas-phase electrostatic interaction (see Table S5†). Hence, we employ the new ALMO-EDA(solv) approach to better capture these interactions in solution. The EDA results for unsubstituted [FeTPP(CO_2_)]^2−^ are shown in Table S4 in the ESI.† This complex is subjected to strong Pauli repulsion (634 kJ mol^−1^), which arises from the repulsion between the iron d-electrons and CO_2_˙^−^ whose excess spin density is mainly located on the carbon atom (see Fig. S4†). The strongly favorable electrostatic interaction (−363 kJ mol^−1^) makes the largest contribution to binding, and is comprised of (i) a moderate gas-phase ELEC term (Δ*E*^(0)^_ELEC_ = −94 kJ mol^−1^) and (ii) a substantially favorable contribution from solute–solvent interaction (Δ*E*_SOL_ = −269 kJ mol^−1^). The former can be rationalized by the attractive short-range Coulomb interaction between the CO_2_˙^−^ moiety and the partially positive-charged iron center, and the latter reflects the solvent screening of the repulsive coulombic interaction between CO_2_˙^−^ and the reduced π system of the TPP ligand. Despite the appreciable electrostatic contribution, the net frozen interaction is still strongly repulsive (+205 kJ mol^−1^), and thus both POL (−135 kJ mol^−1^) and CT (−123 kJ mol^−1^) are essential to the stabilization of CO_2_˙^−^. The electron density difference between the frozen and polarized states reveals that the occupation of iron's d-orbitals changes due to interaction with CO_2_˙^−^. This is mainly to alleviate Pauli repulsion, *via* depopulating the 3d_*z*^2^_ orbital (see Fig. 11(a)). The analysis of complementary occupied-virtual pairs (COVPs)^95^ further demonstrates that the charge transfer in [FeTPP(CO_2_)]^2−^ is dominated by the donation from the odd-electron orbital of CO_2_˙^−^ into the vacant 3d_*z*^2^_ orbital of Fe, where CO_2_˙^−^ acts as a σ-donor as illustrated in Fig. 11(b)).

**Fig. 10:**
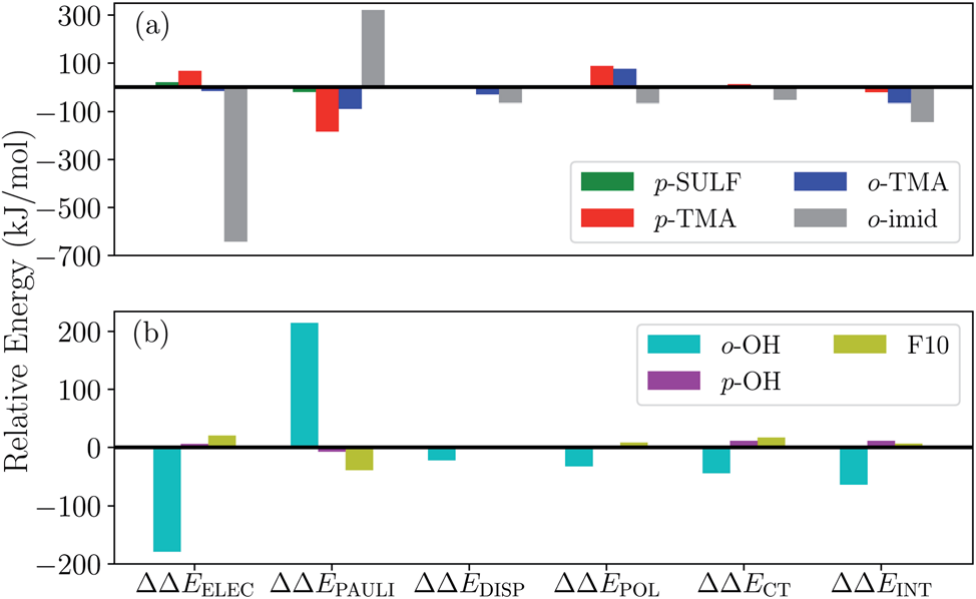
Differential ALMO-EDA(solv) results (in kJ mol^−1^) relative to the unsubstituted [FeTPP(CO_2_)]^2−^ complex: (a) results for the charged substituent groups (–NMe_3_^+^, –SO_3_^−^, and the methylimidazolium-carrying group); (b) results for the substituent groups that retain the net charge of the unsubstituted complex (–OH, –F).

To gauge the effect of the charged substituents (*ortho*- and *para*-TMA and *para*-sulfonate), we compare the total interaction energies and EDA components of these adducts against the results for the unsubstituted [FeTPP(CO_2_-κC)]^2−^. The results are shown in Fig. 10(a). While *para* substitution with sulfonate groups alters the total charge of the CO_2_ adduct from −2 to −4, its effect on the total interaction strength is small. The largest changes in the EDA components occur in the ELEC and PAULI terms: the former becomes slightly less favorable due to the more negatively charged TPP ligand, while the latter is diminished (becoming less unfavorable) by a similar amount, which is possibly related to the weak electron-withdrawing inductive effect of the sulfonate group and the slightly lengthened Fe–C distance in [Fe-*p*-SUL-(CO_2_)]^4−^ (see Table S3†). Besides the changes in ELEC and PAULI that largely cancel each other, the effects of *p*-sulfonate on other energy components (DISP, POL, and CT) are negligible.

**Fig. 11:**
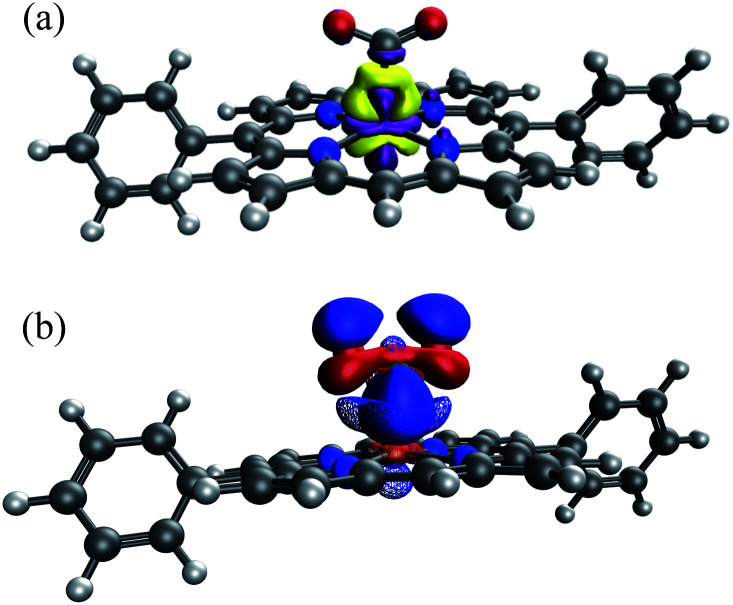
(a) Electron density difference between the FRZ and POL states (yellow: electron density increase, purple: electron density decrease); (b) the key COVPs illustrating the σ donation from the SOMO of CO_2_˙^−^ to Fe's 3d_*z*^2^_ orbital (the donor and acceptor orbitals are plotted with solid and meshed isosurfaces, respectively).

The *p*-TMA group changes the total charge of the CO_2_ adduct from −2 to 0 and strengthens the total interaction by 21 kJ mol^−1^ relative to the unsubstituted FeTPP. Interestingly, the electrostatic interaction is made more repulsive by the *p*-TMA substitution relative to the unsubstituted adduct (by 67 kJ mol^−1^), despite the presence of positively charged TMA groups that can favorably interact with CO_2_˙^−^. Indeed, if one performs EDA in the gas phase, the ELEC term in [Fe-*p*-TMA-(CO_2_)]^0^ is *stabilized* by ∼250 kJ mol^−1^ relative to unsubstituted [FeTPP(CO_2_)]^2−^. However, the electrostatic attraction between the *p*-TMA groups and CO_2_˙^−^ is screened to a large extent in the solvation environment due to the long distance between them (*r*(N⋯O) > 8 Å for N in TMA and O in CO_2_). Complemented by other secondary effects of the strongly electron-withdrawing TMA groups, the ELEC component in fact *destabilizes* [Fe-*p*-TMA-(CO_2_)]^0^ relative to the unsubstituted adduct.

Surprisingly, the largest change among EDA components appears to be the reduction in Pauli repulsion (−183 kJ mol^−1^). This reduction *cannot* be solely explained by the change in the Fe–C distance since its value is almost identical in [Fe-*p*-SUL-(CO_2_)]^4−^ and [Fe-*p*-TMA-(CO_2_)]^0^ (see Table S4†). We attribute it to the decrease in electron density at the iron center, which originates from the weaker coordination of the TPP ligand due to the strong electron-withdrawing inductive effect of the TMA groups and the rigidity of the porphyrin framework that inhibits the further shortening of the Fe–N distances. The polarization term is less favorable in [Fe-*p*-TMA-(CO_2_)]^0^ than that in the unsubstituted adduct, which, however, is a less substantial effect compared to the reduction in Pauli repulsion, and the relative changes in dispersion and charge transfer are of even less significance. Therefore, according to the results of ALMO-EDA(solv), the enhanced CO_2_ stabilization resulting from the *p*-TMA substitution almost entirely arises from the reduction of Pauli repulsion stemming from the strong inductive effect of the TMA group, which is *via* a through-structure rather than through-space mechanism.

Moving the charged TMA groups closer to the CO_2_˙^−^ moiety in [Fe-*o*-TMA-(CO_2_)]^0^ yields more significant relative stabilization than *p*-TMA substitution (65 kJ mol^−1^ relative to the unsubstituted adduct). Surprisingly, the contribution from electrostatic interaction to this relative stabilization is only 16 kJ mol^−1^, which serves as only the third largest contributor. As the distance between CO_2_˙^−^ and TMA is still long in [Fe-*o*-TMA-(CO_2_)]^0^ (*r*(N⋯O) = 3.8 Å for N in TMA and O in CO_2_), the favorable electrostatic interaction remains strongly screened by the (implicit) solvent. As in the *p*-TMA case, the strong inductive effect of the TMA groups reduces Pauli repulsion by 89 kJ mol^−1^, making the largest contribution to the enhanced stabilization. Note that the reduction in the Pauli term here is not as pronounced as that in the *p*-TMA case, which might result from the steric effect of the bulky methyl groups that are in close contact with CO_2_˙^−^. In addition, the Fe–C distance is slightly shorter in the *o*-TMA complex (*r*(Fe–C) = 2.06 Å (*o*-TMA) *vs.* 2.10 Å (*p*-TMA)), which implies a stronger baseline Pauli repulsion. The close contact between methyl groups and CO_2_˙^−^ also results in the strengthened dispersion interaction in the *o*-TMA adduct, which is 30 kJ mol^−1^ more favorable than that in the unsubstituted case and serves as the second largest contributor to the relative stabilization. Combining these factors together, in [Fe-*o*-TMA-(CO_2_)]^0^ we see stabilization of activated CO_2_*via* both through-space (enhanced dispersion and attractive electrostatics) and through-structure (reduction in Pauli repulsion due to the strong inductive effect of TMA) mechanisms, and our EDA results reveal the more significant role of the latter. The co-existence of these two mechanisms leads to larger stabilization of the *o*-TMA substituted adduct.

We next apply our EDA analysis to investigate the stabilization of activated CO_2_ within two adducts whose total charge remains unchanged (−2) upon substitution: the *ortho* hydroxyl substituted adduct [Fe-*o*-OH-(CO_2_)]^2−^ and the F10 derivative. The results are shown in Fig. 10(b). The *o*-OH substitution stabilizes the CO_2_ adduct by 64 kJ mol^−1^, and the EDA results reveal a significantly strengthened electrostatic interaction and moderately increased DISP, POL, and CT components relative to the unsubstituted TPP adduct. Collectively these attractive terms outweigh the increase in Pauli repulsion. A pattern like this is typical of EDA results for hydrogen bonds,^45,46,96^ which in this case are formed between –OH groups at the *ortho* positions of phenyl and the oxygen atoms in CO_2_˙^−^ (see Fig. 9(c)) thanks to the short distance between them (*r*(H⋯O) = 1.79 Å for H in *o*-OH and O in CO_2_). When one moves the hydroxyl group to the *para* position (*p*-OH), no such hydrogen bonds can be formed and consequently there is no notable difference in any of the energy components relative to the unsubstituted adduct. This stark contrast between the results for the *o*-OH and *p*-OH substituted derivatives suggests that the –OH group at the *ortho* position stabilizes CO_2_˙^−^ almost exclusively *via* a through-space mechanism (hydrogen bonding). We note that the stabilization of activated CO_2_ through hydrogen bonds can be further enhanced by precisely tuning the position of H-donor, which was achieved by Nichols *et al.* by introducing amide pendants at the *ortho* position of the meso phenyl groups.^97^ In contrast to [Fe-*o*-OH-(CO_2_)]^2−^, the F10 derivative with only through-structure electron-withdrawing inductive effect does not lead to enhanced stabilization of CO_2_ since the reduction in Pauli repulsion is far less pronounced than that in [Fe-*p*-TMA-(CO_2_)]^0^ or [Fe-*o*-TMA-(CO_2_)]^0^, which is then almost fully compensated by the diminished ELEC and CT contributions.

**Fig. 12:**
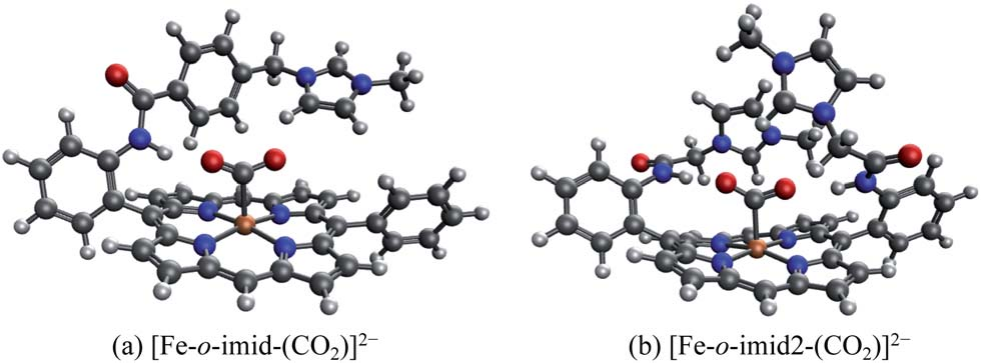
Optimized geometries for (a) [Fe-*o*-imid-(CO_2_)]^−^, which carries one methylimidazolium-containing group that was reported in ref. 98, and (b) [Fe-*o*-imid2-(CO_2_)]^2−^, which carries two modified methylimidazolium-containing groups.

The EDA results for the TMA-substituted derivatives suggest that the strategy to stabilize activated CO_2_ through long-range electrostatic attraction may not be fully effective in solution due to solvent screening. However, making use of the steric effects of the substituents, one may be able to create a solvent-free “pocket” in which electrostatic interaction is almost unscreened. It was reported by Khadhraoui *et al.* that the introduction of four bulky, methylimidazolium-containing groups at the *ortho* positions of the phenyl groups in FeTPP elevates its electrocatalytic activity.^98^ Due to the substantial size of the substituent, we optimized the structure of the CO_2_ adduct of this FeTPP derivative with only one methylimidazolium-containing “arm” included (reducing the negative charge from −2 to −1), which is denoted as [Fe-*o*-imid-(CO_2_)]^−^. The optimized structure of this adduct is depicted in Fig. 12(a). Differing from [Fe-*o*-TMA-(CO_2_)]^0^, the charged moiety (imidazolium ring) is far away from the central metal, excluding the possibility of any electron-withdrawing inductive effect from this positively charged substituent. A remarkable feature of the optimized structure is that this “long-arm” substituent folds over the activated CO_2_ and thereby creating a small pocket that is inaccessible by solvent. The geometry also demonstrates that the activated CO_2_ is stabilized by both the hydrogen bonding from the amide group and the electrostatic attraction from the positively charged methylimidazolium moiety: the H⋯O distance in this hydrogen bond is 1.98 Å, and the distance between the mid-point of two N atoms in the imidazolium ring and the closest O atom in CO_2_ is 4.55 Å.

Our results show that [Fe-*o*-imid-(CO_2_)]^−^ stabilizes CO_2_ more strongly by −49.2 kJ mol^−1^ relative to the unsubstituted FeTPP (see Table S4 in the ESI†), similar to the *o*-OH and *o*-TMA cases. However, it should be noted that here we only included one substituent group (“arm”) whereas the other derivatives are doubly substituted in our calculations. Therefore, once the two methylimidazolium-containing “arms” are both included, one can expect that this complex can stabilize activated CO_2_ more than the *o*-OH and *o*-TMA derivatives. The ALMO-EDA(solv) results (Table S4†) show that the dominant contributor to stabilization is electrostatic interaction (−85.9 kJ mol^−1^), which stems from both the N–H⋯O hydrogen bond and the positively charged methylimidazolium. The key difference from the *p*-TMA substituent is that this bulky ligand effectively “squeezes out” solvent from the space in between the activated CO_2_ and the positively charged moiety, rendering the attractive electrostatics nearly unscreened. Furthermore, as in the *o*-OH case, the strength of dispersion, polarization, and charge transfer is also enhanced by the introduction of this methylimidazolium-containing substituent, which, together with the gain in attractive electrostatics, contribute to this more stabilized CO_2_ adduct.

In order to further optimize this interaction motif, we removed the phenyl group in the methylimidazolium-containing “arm”, which moves the methylimidazolium moiety closer to the CO_2_ moiety. The optimized structure for this adduct (denoted as [Fe-*o*-imid2-(CO_2_)]^0^ since the second substituent is added for a better comparison with the results of other derivatives) is shown in Fig. 12(b), in which the distance between the mid-point of two N atoms in the imidazolium ring and the closest O atom in CO_2_ reduces to 3.74 Å. The stabilization energy relative to the unsubstituted adduct is −143.5 kJ mol^−1^, which is significantly larger than that of the second most stabilized *o*-TMA and *o*-OH derivatives (see Fig. 10(a)). The relative stabilization arising from the electrostatic attraction is *by far* the strongest among the adducts that we investigated (−642.0 kJ mol^−1^), and we estimate that −140 kJ mol^−1^ out of that stems from the two hydrogen bonds (see the “NH-ref” results in Table S4†) and the rest (about −500 kJ mol^−1^) from the imidazolium rings. These results indicate the importance of taking solvent effects into account if one wants to harness through-space Coulomb interaction in solution and pursue the promise of bulky, flexible substituents that can form a solvent-free “active” pocket mimicking that in enzymes to facilitate much stronger electrostatic interaction with CO_2_.

It is important to point out that we focused here on the concept of strengthening the binding of activated CO_2_ in adducts as that was presumed to accelerate catalysis. However, the substituents may also affect other intermediates in a catalytic cycle. This is illustrated by the *ortho* hydroxyl substituted TPP complex, CAT, where the hydroxyl group stabilizes the activated CO_2_ and also leads to a fast intramolecular protonation pathway.^58^ Ultimately, detailed mechanistic studies are necessary to fully unravel the influence of each substituent on the catalytic activity.

### 4.4 Electron transfer from terphenyl˙^−^ to CO_2_: substituent effects on the intermolecular binding of reactant and product complexes

An alternative to transition metal based catalysts for CO_2_ reduction is organic (photo)redox catalysts, which can be more environment-friendly, economical, and are likewise highly tunable with substituents.^99^ These catalysts access their electronic excited states through UV-Vis absorption and are subsequently quenched, yielding a reactive radical species that serves as the electron donor to CO_2_.^99,100^ A prominent class of examples are oligo(*p*-phenylenes) (OPPs), which, for instance, are able to catalyze hydrocarboxylation from CO_2_.^53^ The introduction of different substituents alters the absorption wavelength but also impacts the rate of electron transfer. Some of us recently^101^ investigated the substituent effects on the calculated rate of electron transfer (ET) reaction from an OPP radical anion to molecular CO_2_ using double terminal-substituted *p*-terphenyl as examples. We have shown that electron-donating groups (EDGs) facilitate this reaction in general by increasing the free energy driving force (Δ*G* in Marcus theory^102^) since they elevate the LUMO level of OPP. Besides the reductive potential of OPP/OPP˙^−^, the difference in the association energies of the reactant (OPP˙^−^⋯CO_2_) and product (OPP⋯CO_2_˙^−^) complexes in solution, ΔΔ*E*_INT_ = Δ*E*_INT_(**R**_r_) − Δ*E*_INT_(**R**_p_), also contributes to the free energy driving force (**R**_r_ and **R**_p_ denote optimized structures for the reactant and product states, respectively). Here we employ ALMO-EDA(solv) to investigate the substituent effects on the association energies of the reactant and product complexes in CH_2_Cl_2_ (*ε* = 8.93) described by the SMD model, which will afford us deeper insight into how these chemical modifications affect the reactivity of OPPs as photoredox catalysts.

**Fig. 13:**
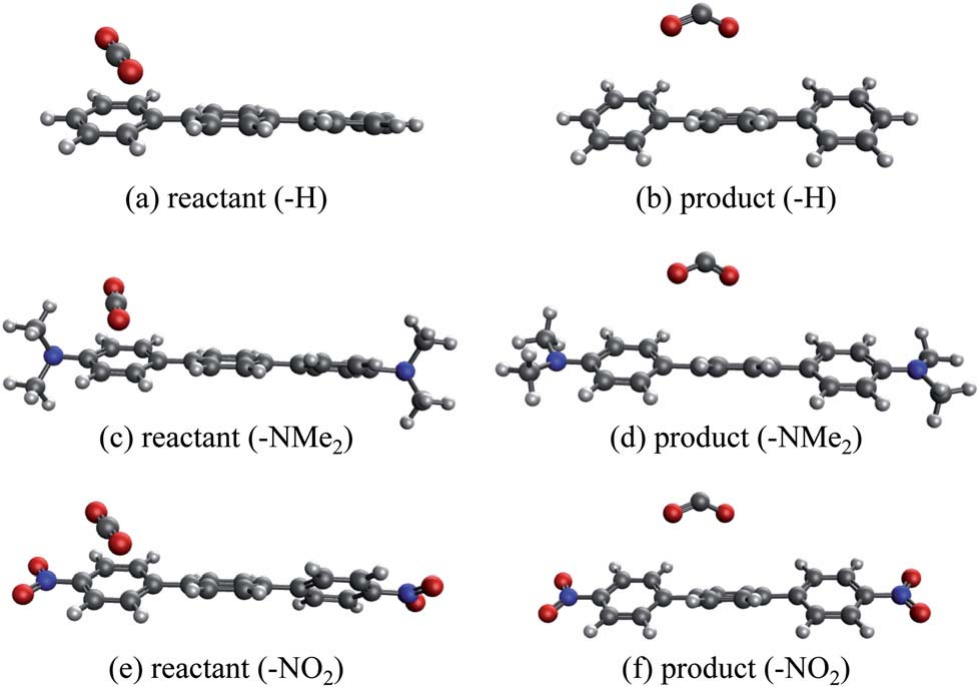
Optimized structures of unsubstituted and substituted *p*-terphenyl⋯CO_2_ radical anion complexes: (a and b) unsubstituted reactant and product complexes; (c and d) NMe_2_-substituted; (e and f) NO_2_-substituted.

The geometries of the reactant and product complexes are directly taken from our previous work,^101^ which were optimized on their respective diabatic PESs constructed from constrained DFT (CDFT)^103,104^ calculations at the B3LYP-D3(BJ)/6-311G(d,p)^105–107^ level of theory with C-PCM. As illustrative examples, in Fig. 13 we show the optimized structures of the reactant and product complexes between CO_2_ (or CO_2_˙^−^) and *p*-terphenyl˙^−^ (or neutral *p*-terphenyl) substituted with dimethylamino (–NMe_2_) and nitro (–NO_2_) groups. Compared to the reactant complexes where CO_2_ is linear and mainly interacting with one of the terminal phenyl groups, in the product state the CO_2_ moiety is bent and moves closer to the middle ring of *p*-terphenyl. By contrast with the adducts with FeTPP where CO_2_˙^−^ is ligated to Fe through the κC mode (see Section 4.3), here in the product state the two oxygen atoms of CO_2_˙^−^ are in closer contact with the *p*-terphenyl moiety, an orientation that is favored by these dispersion-dominated anion-π interactions (*vide infra*).

**Fig. 14:**
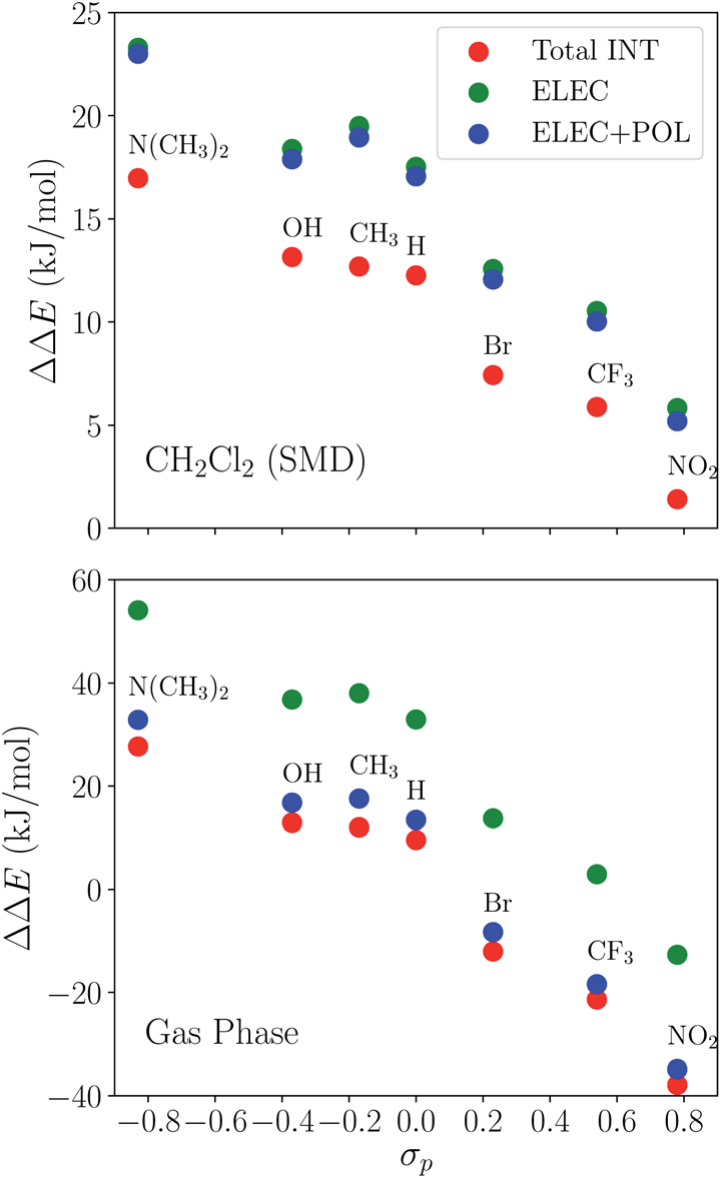
Substituent effects on the differences in total interaction energies as well as the ELEC and POL components (in kJ mol^−1^) between the reactant and product states. The calculations are performed at the *ω*B97X-V/def2-TZVPD level of theory. The upper panel shows results in dichloromethane (CH_2_Cl_2_) solvent described by SMD and the lower panel shows gas-phase results. The *x*-axis shows the Hammett parameter of each substituent group.

The substituent effect on the difference between interaction energies in the reactant and product states (ΔΔ*E*_INT_) in CH_2_Cl_2_ solution is exhibited in the upper panel of Fig. 14 with three electron-donating (–NMe_2_, –OH, –CH_3_) and three electron-withdrawing (–Br, –CF_3_, –NO_2_) groups. The strength of the electronic effects of these substituent groups can be characterized using their Hammett parameters^108^ (*σ*_p_): more negative (positive) *σ*_p_ indicates stronger electron-donating (withdrawing) ability. It is shown that the difference in interaction energies decreases monotonically with increases in *σ*_p_, *i.e.*, strong electron-withdrawing groups (EWGs) such as –NO_2_ facilitate the stabilization of the product complex relative to the reactant. Relative to the unsubstituted species, the differential product stabilization by the strongest EWG, –NO_2_, is over 10 kJ mol^−1^. ALMO-EDA(solv) reveals that this prominent substituent effect is dominated by electrostatics despite the presence of solvent (see Table S6 in ESI† for the full EDA results). As shown in the left panel of Fig. 14, ΔΔ*E*_ELEC_ reproduces the trend in ΔΔ*E*_INT_ with only one marginal exception (–CH_3_).

To demonstrate the effect of solvent on the trend in ΔΔ*E*_INT_*versus σ*_p_, we also performed ALMO-EDA calculations for the same set of complexes in vacuum (see Table S7 in ESI† for the complete results). The results for the total interaction energy and the ELEC component are shown in the lower panel of Fig. 14. While the same trend in ΔΔ*E*_INT_ (monotonically decrease with increasing *σ*_p_) is reproduced without solvent, the magnitude of ΔΔ*E*_INT_ with different substituent groups exhibits a much wider range, which, once again, mainly results from the larger variation in ΔΔ*E*_ELEC_ in vacuum. In contrast to the solution phase where the polarization component exhibits only minimal effects on ΔΔ*E*_INT_, in the gas phase, POL stabilizes the product complex by ∼20 kJ mol^−1^ relative to the reactant for these complexes. The contrast between the upper and lower panels of Fig. 14 demonstrates the *attenuation* of substituent effects on the differential interaction energies due to solvent screening.

In our previous study^101^ where the ALMO-EDA calculations were performed in vacuum at the B3LYP-D3(BJ)/6-311G(d,p) level of theory, we identified CT as another main contributor to the stabilization of the product complexes relative to the reactant ones and also to the trend in substituent effects. This contradicts the gas-phase ALMO-EDA results obtained here with *ω*B97X-V/def2-TZVPD, according to which CT only makes a minimal contribution to each complex's ΔΔ*E*_INT_ (see the comparison between Tables S7 and S8 in the ESI†). We ascribe this discrepancy to the more substantial delocalization error^109,110^ associated with the B3LYP functional than that of *ω*B97X-V, which, as shown in Table S10 in the ESI,† has a more pronounced effect in the gas phase. We refer the reader to Section S3 in the ESI† for a detailed discussion.

**Fig. 15:**
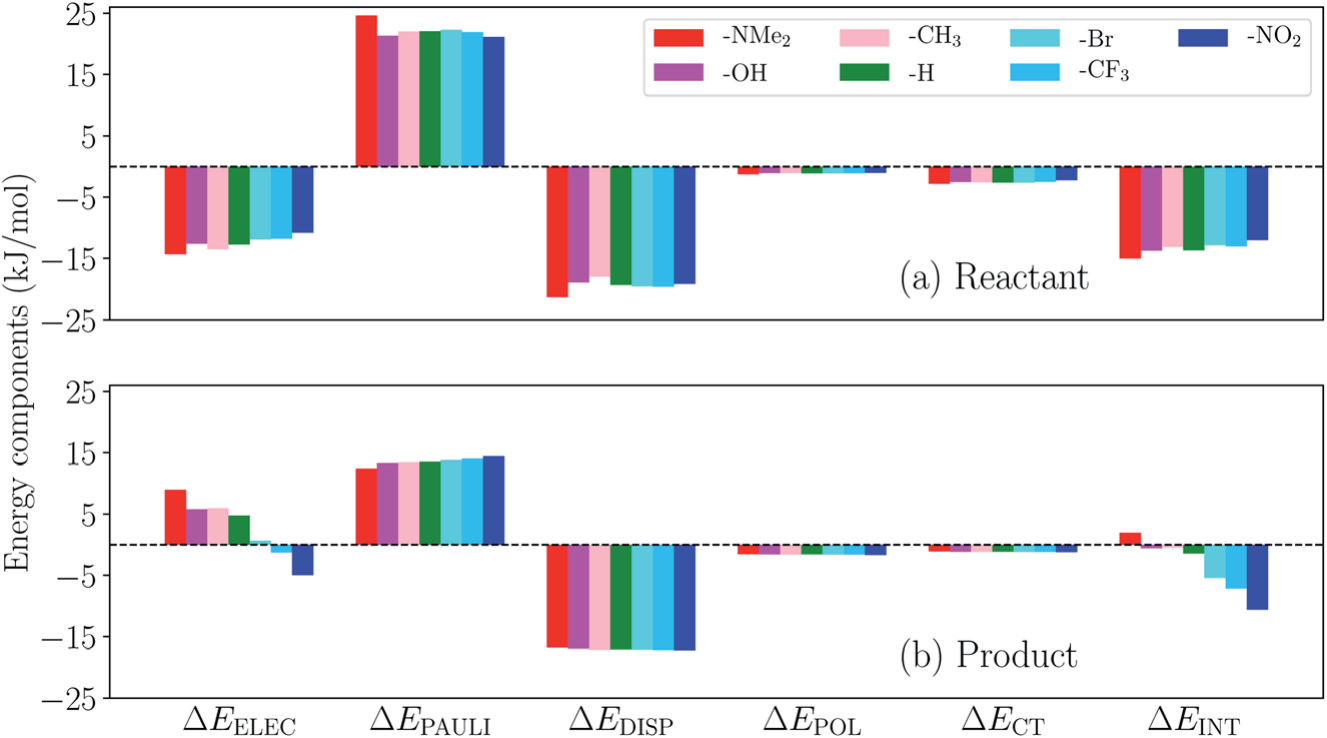
ALMO-EDA(solv) results (in kJ mol^−1^) for the (a) reactant and (b) product complexes between *p*-terphenyl and CO_2_ (one of these two species carries −1 charge) with different substituent groups.

We then turn to the full ALMO-EDA(solv) results for the reactant and product complexes to gain further insights into the substituent effects on ΔΔ*E*_INT_ revealed in Fig. 14. The reactant complexes (Fig. 15(a)) are mainly bound by electrostatic and dispersion interactions, which, taken together, overcome the Pauli repulsion between *p*-terphenyl˙^−^ (or its substituted derivatives) and the neutral CO_2_ moiety. Moderate substituent effects are exhibited among the reactant complexes, where the EDGs yield more attractive total interactions in general (*e.g.* the NMe_2_-substituted complex is more favorable than NO_2_-substituted by ∼3 kJ mol^−1^). This trend, as revealed by the EDA results, mainly stems from the enhancement in electrostatic interaction with stronger electron-donating substituents. The same trend is also exhibited in the strength of POL and CT across different substituents, despite the relatively small magnitude of these two components. The product complexes, on the other hand, are not as strongly bound as in their respective reactant state, and they are mainly stabilized by dispersion interaction according to our EDA results (Fig. 15(b)). The magnitude of the total interaction is strongly impacted by the substituent group on the *p*-terphenyl moiety: with EDGs (–NMe_2_, –OH, and –CH_3_), the intermolecular binding between terphenyl and CO_2_˙^−^ is of only minimal strength (less favorable than −1 kJ mol^−1^), while with EWGs (–Br, –CF_3_, and –NO_2_) the interaction becomes increasingly more favorable with the increase in substituent's electron-withdrawing ability (*σ*_p_). Note that the resulting interaction energy for the NMe_2_-substituted product complex is net repulsive (+1.96 kJ mol^−1^), which most likely arises from the distinct levels of theory that were used in our previous CDFT geometry optimizations^101^ and the ALMO-EDA(solv) calculations in the present paper. Similar to the reactant complexes, the substituent effects on the total interaction strength in the product state is also dominated by the ELEC component, where the EWGs are shown to strengthen the binding by reducing the electrostatic repulsion between CO_2_˙^−^ and the π electrons on the *p*-terphenyl moiety.

To summarize, the substituent effects on the intermolecular binding strength of the reactant and product *p*-terphenyl⋯CO_2_ radical anion complexes exhibit opposite trends, where EWGs diminish and enhance the interaction in the reactant and product states, respectively. While dispersion contributes significantly to binding in both states, the substituent effects are mainly controlled by the electrostatic component in both states despite the presence of solvent environment. These two opposite trends, combined together, lead to the trend shown in Fig. 14 where EWGs yield more strongly bound product complexes relative to the reactant ones. Interestingly, the trend in ΔΔ*E*_INT_ with respect to varying *σ*_p_ is opposite to that in the total free energy change (Δ*G*) upon the electron transfer (ET), where EDGs yield more favorable driving forces.^101^ This implies that although EWGs assist in stabilizing the product complexes, they are unable to reverse the trend in the free energy driving force dominated by the gap between the monomer energies before and after ET. In Table S11 in the ESI,† we show the comparison between ΔΔ*E*_INT_ and the energy associated with the reactant-to-product electronic transitions at the monomer level (denoted as Δ*E*_PREP_) for this series of complexes.

## 5 Conclusions

In this work, we have developed the ALMO-EDA(solv) scheme to incorporate solvation effects described by dielectric continuum models into the second-generation ALMO-EDA based on DFT calculations.^46^ This method possesses the following main features:

(1) The implicit solvent environment is included in the construction of all states across the EDA procedure. Hence all energy differences (Δ*E*_FRZ_, Δ*E*_POL_, and Δ*E*_CT_) are always computed between two consecutive states that are both properly solvated.

(2) A new term, Δ*E*_SOL_, is introduced to describe the direct change in solute–solvent interaction energy upon the formation of the frozen complex. In most generic cases, it comprises both electrostatic and non-electrostatic components, which can further be combined into the ELEC and PAULI components of the (internal) frozen interaction energy.

ALMO-EDA(solv) consistently incorporates continuum solvent effects, which permits study of solvation effects on each energy component in a systematic, physically motivated manner. To validate our EDA scheme, we first investigated the electrostatics-related terms using a solvated Na^+^⋯Cl^−^ model complex. Our EDA reproduces the correct bulk limit for long-range electrostatics in solvent. We also rationalized diminished screening in the short range. We next analyzed the distance dependence of the energy components produced by ALMO-EDA(solv) for the H_2_O⋯Na^+^ and H_2_O⋯Cl^−^ complexes and demonstrated how solvents with varying dielectric constants affect the net strength of permanent electrostatics and polarization in these systems. The results further confirmed that ALMO-EDA(solv) yields physically sensible results for the energy components of these simple interactions in the solution phase and correctly reflects the trend in the relative strength of solvent effect.

We then employed ALMO-EDA(solv) to investigate more complex chemical systems related to catalysis of CO_2_ reduction reactions. We first considered CO_2_ complexes with doubly reduced FeTPP (TPP = tetraphenylporphyrin) and its substituted derivatives.^51,90^ We found that the most strongly bound *o*-TMA-substituted complex is not mainly stabilized *via* through-space electrostatic attraction as was presumed (since the solvation environment screens this interaction significantly). Instead, it mainly benefits from reduced Pauli repulsion compared to the unsubstituted [FeTPP(CO_2_-κC)]^2−^ complex. This originates from the substantial electron-withdrawing inductive effect of the positively charged trimethylammonio groups that reduces electron density around the Fe center and give rise to a more Lewis acidic metal center. This stabilization is thus *via* a through-structure mechanism. Another strongly bound complex with *o*-OH substitution, in contrast, is mainly stabilized *via* hydrogen bonding between the *o*-OH groups and the negatively charged CO_2_ moiety, which is exclusively a through-space effect. Our study thus provides new insights into how substituent effects influence the ability of FeTPP to stabilize activated CO_2_. Inspired by the EDA results and the ligand reported in ref. 98, we designed a bulky, floppy substituent group that contains a positively charged methylimidazolium moiety. When introduced to the *ortho* positions of the phenyl groups in FeTPP, they can create a solvent-inaccessible “pocket” in that stabilization of activated CO_2_*via* long-range Coulomb interaction can be achieved due to the removal of solvent screening effects.

Second, we investigated complexes associated with the electron transfer reaction from *p*-terphenyl˙^−^ (and its double terminal-substituted derivatives) to CO_2_. We demonstrated that differences between the interaction energies in the reactant and product states (ΔΔ*E*_INT_) are considerably modulated by the substituents, where electron-withdrawing groups were shown to stabilize the product complexes while moderately destabilizing the ones in the reactant state. Our EDA results further revealed that although dispersion plays an important role in the formation of both reactant and product complexes, the substituent tuning of ΔΔ*E*_INT_ is almost entirely achieved through modulating the electrostatic component (Δ*E*_ELEC_) especially that in the product state. This example shows how ALMO-EDA(solv) assists in elucidating the nature of intermolecular interactions and mechanisms of chemical processes in the solution phase.

Finally, we shall note some of the limitations of the present ALMO-EDA(solv) scheme. First, as we have noted in Section 2, currently available DFT-based EDA schemes including our approach are most likely unable to fully describe the many-body solvent effect on dispersion interactions. Second, the current ALMO-EDA(solv) scheme is limited to the decomposition of single-point (*vertical*) interaction energies, and it is certainly desirable to further extend its capability to enable analysis of molecular property shifts in solution based upon our previously developed *adiabatic* ALMO-EDA scheme.^111^ This would require the development of nuclear gradients for the FRZ and POL intermediate states in the presence of implicit solvent. Besides these limitations from the perspective of EDA, one should also bear in mind that there are many other molecular approaches to describe solvent effects in modern theoretical chemistry that are more sophisticated than the relatively simple dielectric continuum model. It is an interesting challenge to make an EDA scheme compatible with those more advanced solvation models. These limitations, on the other hand, provide a wide range of future opportunities to further extend the treatment of solvation effects in ALMO-EDA calculations.

## Conflicts of interest

The authors declare the following competing financial interest(s): M. H.-G. is a part-owner of Q-Chem Inc.

## Supplementary Material

SC-012-D0SC05327A-s001

SC-012-D0SC05327A-s002

SC-012-D0SC05327A-s003

SC-012-D0SC05327A-s004

SC-012-D0SC05327A-s005

SC-012-D0SC05327A-s006

SC-012-D0SC05327A-s007

SC-012-D0SC05327A-s008

SC-012-D0SC05327A-s009

SC-012-D0SC05327A-s010

SC-012-D0SC05327A-s011

SC-012-D0SC05327A-s012

SC-012-D0SC05327A-s013

SC-012-D0SC05327A-s014

SC-012-D0SC05327A-s015

SC-012-D0SC05327A-s016

SC-012-D0SC05327A-s017

SC-012-D0SC05327A-s018

SC-012-D0SC05327A-s019

SC-012-D0SC05327A-s020

SC-012-D0SC05327A-s021

SC-012-D0SC05327A-s022

SC-012-D0SC05327A-s023

SC-012-D0SC05327A-s024

SC-012-D0SC05327A-s025

SC-012-D0SC05327A-s026

SC-012-D0SC05327A-s027

SC-012-D0SC05327A-s028

SC-012-D0SC05327A-s029

SC-012-D0SC05327A-s030

SC-012-D0SC05327A-s031

SC-012-D0SC05327A-s032

SC-012-D0SC05327A-s033

SC-012-D0SC05327A-s034

SC-012-D0SC05327A-s035

SC-012-D0SC05327A-s036

SC-012-D0SC05327A-s037

SC-012-D0SC05327A-s038

SC-012-D0SC05327A-s039

SC-012-D0SC05327A-s040

SC-012-D0SC05327A-s041

SC-012-D0SC05327A-s042

SC-012-D0SC05327A-s043

SC-012-D0SC05327A-s044

SC-012-D0SC05327A-s045

SC-012-D0SC05327A-s046

SC-012-D0SC05327A-s047

SC-012-D0SC05327A-s048

SC-012-D0SC05327A-s049

SC-012-D0SC05327A-s050

SC-012-D0SC05327A-s051

SC-012-D0SC05327A-s052

SC-012-D0SC05327A-s053

SC-012-D0SC05327A-s054

SC-012-D0SC05327A-s055

SC-012-D0SC05327A-s056

SC-012-D0SC05327A-s057

SC-012-D0SC05327A-s058

SC-012-D0SC05327A-s059

SC-012-D0SC05327A-s060

SC-012-D0SC05327A-s061

SC-012-D0SC05327A-s062

SC-012-D0SC05327A-s063

SC-012-D0SC05327A-s064

SC-012-D0SC05327A-s065

SC-012-D0SC05327A-s066

SC-012-D0SC05327A-s067

SC-012-D0SC05327A-s068

SC-012-D0SC05327A-s069

SC-012-D0SC05327A-s070

SC-012-D0SC05327A-s071

SC-012-D0SC05327A-s072

SC-012-D0SC05327A-s073

SC-012-D0SC05327A-s074

SC-012-D0SC05327A-s075

SC-012-D0SC05327A-s076

SC-012-D0SC05327A-s077

SC-012-D0SC05327A-s078

SC-012-D0SC05327A-s079

SC-012-D0SC05327A-s080

SC-012-D0SC05327A-s081

SC-012-D0SC05327A-s082

SC-012-D0SC05327A-s083

SC-012-D0SC05327A-s084

SC-012-D0SC05327A-s085

SC-012-D0SC05327A-s086

SC-012-D0SC05327A-s087

SC-012-D0SC05327A-s088

SC-012-D0SC05327A-s089

SC-012-D0SC05327A-s090

SC-012-D0SC05327A-s091

SC-012-D0SC05327A-s092

SC-012-D0SC05327A-s093

SC-012-D0SC05327A-s094

SC-012-D0SC05327A-s095

SC-012-D0SC05327A-s096

SC-012-D0SC05327A-s097

SC-012-D0SC05327A-s098

SC-012-D0SC05327A-s099

SC-012-D0SC05327A-s100

SC-012-D0SC05327A-s101

SC-012-D0SC05327A-s102

SC-012-D0SC05327A-s103

SC-012-D0SC05327A-s104

SC-012-D0SC05327A-s105

SC-012-D0SC05327A-s106

SC-012-D0SC05327A-s107

SC-012-D0SC05327A-s108

SC-012-D0SC05327A-s109

SC-012-D0SC05327A-s110

SC-012-D0SC05327A-s111

SC-012-D0SC05327A-s112

SC-012-D0SC05327A-s113

SC-012-D0SC05327A-s114

SC-012-D0SC05327A-s115

SC-012-D0SC05327A-s116

SC-012-D0SC05327A-s117

SC-012-D0SC05327A-s118

SC-012-D0SC05327A-s119

SC-012-D0SC05327A-s120

SC-012-D0SC05327A-s121

SC-012-D0SC05327A-s122

SC-012-D0SC05327A-s123

SC-012-D0SC05327A-s124

SC-012-D0SC05327A-s125

SC-012-D0SC05327A-s126

SC-012-D0SC05327A-s127

SC-012-D0SC05327A-s128

SC-012-D0SC05327A-s129

SC-012-D0SC05327A-s130

SC-012-D0SC05327A-s131

SC-012-D0SC05327A-s132

SC-012-D0SC05327A-s133

SC-012-D0SC05327A-s134

SC-012-D0SC05327A-s135

SC-012-D0SC05327A-s136

SC-012-D0SC05327A-s137

SC-012-D0SC05327A-s138

SC-012-D0SC05327A-s139

SC-012-D0SC05327A-s140

SC-012-D0SC05327A-s141

SC-012-D0SC05327A-s142

SC-012-D0SC05327A-s143

SC-012-D0SC05327A-s144

SC-012-D0SC05327A-s145

SC-012-D0SC05327A-s146

SC-012-D0SC05327A-s147

SC-012-D0SC05327A-s148

SC-012-D0SC05327A-s149

SC-012-D0SC05327A-s150

SC-012-D0SC05327A-s151

SC-012-D0SC05327A-s152

SC-012-D0SC05327A-s153

SC-012-D0SC05327A-s154

SC-012-D0SC05327A-s155

SC-012-D0SC05327A-s156

SC-012-D0SC05327A-s157

SC-012-D0SC05327A-s158

SC-012-D0SC05327A-s159

SC-012-D0SC05327A-s160

SC-012-D0SC05327A-s161

SC-012-D0SC05327A-s162

SC-012-D0SC05327A-s163

SC-012-D0SC05327A-s164

SC-012-D0SC05327A-s165

SC-012-D0SC05327A-s166

SC-012-D0SC05327A-s167

SC-012-D0SC05327A-s168

SC-012-D0SC05327A-s169

SC-012-D0SC05327A-s170

SC-012-D0SC05327A-s171

SC-012-D0SC05327A-s172

SC-012-D0SC05327A-s173

SC-012-D0SC05327A-s174

SC-012-D0SC05327A-s175

SC-012-D0SC05327A-s176

SC-012-D0SC05327A-s177

SC-012-D0SC05327A-s178

SC-012-D0SC05327A-s179

SC-012-D0SC05327A-s180

SC-012-D0SC05327A-s181

SC-012-D0SC05327A-s182

SC-012-D0SC05327A-s183

SC-012-D0SC05327A-s184

SC-012-D0SC05327A-s185

SC-012-D0SC05327A-s186

SC-012-D0SC05327A-s187

SC-012-D0SC05327A-s188

SC-012-D0SC05327A-s189

SC-012-D0SC05327A-s190

SC-012-D0SC05327A-s191

SC-012-D0SC05327A-s192

SC-012-D0SC05327A-s193

SC-012-D0SC05327A-s194

SC-012-D0SC05327A-s195

SC-012-D0SC05327A-s196

SC-012-D0SC05327A-s197

SC-012-D0SC05327A-s198

SC-012-D0SC05327A-s199

SC-012-D0SC05327A-s200

SC-012-D0SC05327A-s201

SC-012-D0SC05327A-s202

SC-012-D0SC05327A-s203

SC-012-D0SC05327A-s204

SC-012-D0SC05327A-s205

SC-012-D0SC05327A-s206

SC-012-D0SC05327A-s207

SC-012-D0SC05327A-s208

SC-012-D0SC05327A-s209

SC-012-D0SC05327A-s210

SC-012-D0SC05327A-s211

SC-012-D0SC05327A-s212

SC-012-D0SC05327A-s213

SC-012-D0SC05327A-s214

SC-012-D0SC05327A-s215

SC-012-D0SC05327A-s216

SC-012-D0SC05327A-s217

SC-012-D0SC05327A-s218

SC-012-D0SC05327A-s219

SC-012-D0SC05327A-s220

SC-012-D0SC05327A-s221

SC-012-D0SC05327A-s222

SC-012-D0SC05327A-s223

SC-012-D0SC05327A-s224

SC-012-D0SC05327A-s225

SC-012-D0SC05327A-s226

SC-012-D0SC05327A-s227

SC-012-D0SC05327A-s228

SC-012-D0SC05327A-s229

SC-012-D0SC05327A-s230

SC-012-D0SC05327A-s231

SC-012-D0SC05327A-s232

SC-012-D0SC05327A-s233

SC-012-D0SC05327A-s234

SC-012-D0SC05327A-s235

SC-012-D0SC05327A-s236

SC-012-D0SC05327A-s237

SC-012-D0SC05327A-s238

SC-012-D0SC05327A-s239

SC-012-D0SC05327A-s240

SC-012-D0SC05327A-s241

SC-012-D0SC05327A-s242

SC-012-D0SC05327A-s243

SC-012-D0SC05327A-s244

SC-012-D0SC05327A-s245

SC-012-D0SC05327A-s246

SC-012-D0SC05327A-s247

SC-012-D0SC05327A-s248

SC-012-D0SC05327A-s249

SC-012-D0SC05327A-s250

SC-012-D0SC05327A-s251

SC-012-D0SC05327A-s252

SC-012-D0SC05327A-s253

SC-012-D0SC05327A-s254

SC-012-D0SC05327A-s255

SC-012-D0SC05327A-s256

SC-012-D0SC05327A-s257

SC-012-D0SC05327A-s258

SC-012-D0SC05327A-s259

SC-012-D0SC05327A-s260

SC-012-D0SC05327A-s261

SC-012-D0SC05327A-s262

SC-012-D0SC05327A-s263

SC-012-D0SC05327A-s264

SC-012-D0SC05327A-s265

SC-012-D0SC05327A-s266

SC-012-D0SC05327A-s267

SC-012-D0SC05327A-s268

SC-012-D0SC05327A-s269
