# Supplementary material for: Consistent inclusion of continuum solvation in energy decomposition analysis: theory and application to molecular CO2 reduction catalysts
Source: Chem Sci. 2020 Nov 27;12(4):1398–414. doi: 10.1039/d0sc05327a (PMC8179122; doi:10.1039/d0sc05327a)
Supplement: SC-012-D0SC05327A-s001 [file SC-012-D0SC05327A-s001.pdf]

Electronic Supplementary Material (ESI) for Chemical Science.
This journal is © The Royal Society of Chemistry 2020

Electronic Supplementary Information for "Consistent Inclusion of Continuum Solvation in Energy Decomposition Analysis: Theory and Application to Molecular CO_2 Reduction Catalysts"

Yuezhi Mao,*[a‡§] Matthias Loipersberger,[a‡] Kareesa J. Kron,[b] Jeffrey S. Derrick,[a] Christopher J. Chang,[acd], Shaama Mallikarjun Sharada,[be] and Martin Head-Gordon*[ac]

November 26, 2020

Contents

S1 Additional results for the validation of ALMO-EDA(solv) **2**

S2 Additional results for the [FeTPP($CO_2^{\bullet-}$)] derivatives **4**

S3 Additional results for the substituted terphenyl$\cdots CO_2$ complexes **6**

S4 Sample input for calculations using PCM **11**

[a] *Department of Chemistry, University of California at Berkeley, Berkeley, CA 94720, USA; E-mail: yuezhi.mao@berkeley.edu, mhg@cchem.berkeley.edu*
[b] *Mork Family Department of Chemical Engineering and Material Science, University of Southern California, Los Angeles, CA 90089, USA*
[c] *Chemical Sciences Division, Lawrence Berkeley National Laboratory, Berkeley, CA 94720, USA*
[d] *Department of Molecular and Cell Biology, University of California Berkeley, Berkeley, CA 94720, USA*
[e] *Department of Chemistry, University of Southern California, Los Angeles, CA 90089, USA*
‡ These two authors contributed equally
§ Present Address: Department of Chemistry, Stanford University, Stanford, CA 94305, USA

S1 Additional results for the validation of ALMO-EDA(solv)

Table S1: Strength (in kJ/mol) of internal QM electrostatics ($\Delta E^{(0)}_{\rm ELEC}$) and the effect of solute-solvent electrostatic interaction on binding ($\Delta E^{\rm el}_{\rm SOL}$) for $Na^{+}\cdots Cl^{-}$ separated by 20 Å calculated with ωB97X-V/def2-TZVPPD and IEF-PCM with varying dielectric constants. $\Delta E^{(s)}_{\rm ELEC} = \Delta E^{(0)}_{\rm ELEC} + \Delta E^{\rm el}_{\rm SOL}$ is the effective (screened) electrostatic interaction in the solution phase.

ϵ	$\Delta E^{(0)}_{\rm ELEC}$	$\Delta E^{\rm el}_{\rm SOL}$	$\Delta E^{(s)}_{\rm ELEC}$	$\Delta E^{(0)}_{\rm ELEC}/\Delta E^{(s)}_{\rm ELEC}$
1	-69.47	0	-69.47	1.0
10	-69.47	62.52	-6.94	10.0
20	-69.47	65.99	-3.47	20.0
40	-69.47	67.73	-1.74	40.0
80	-69.47	68.60	-0.87	79.9

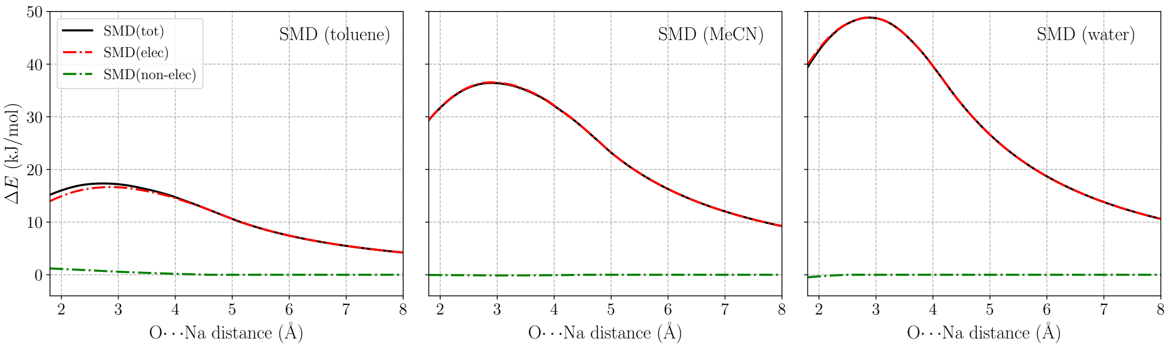

Figure S1: Electrostatic and non-electrostatic components (in kJ/mol) of the solvent contribution ($\Delta E_{\rm SOL}$) to the $H_2O\cdots Na^{+}$ interaction with the $O\cdots Na^{+}$ distance ranging from 1.8 to 8.0 Å, with solvents toluene, MeCN, and water modeled by SMD.

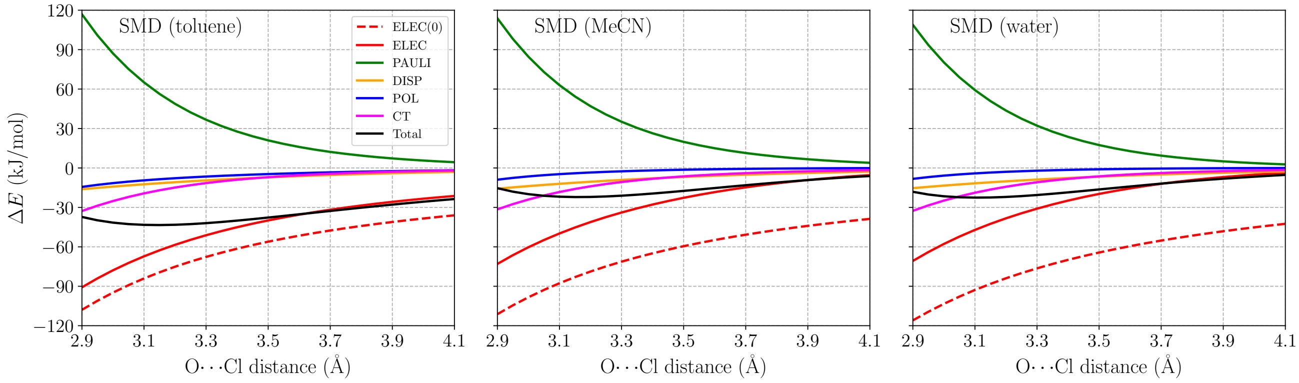

Figure S2: ALMO-EDA(sol) results (in kJ/mol) for the $H_2O\cdots Cl^-$ complex in toluene, acetonitrile (MeCN), and water solutions with the $O\cdots Cl^-$ distance ranging from 2.9 to 4.1 Å. All calculations are performed using ωB97X-V/def2-TZVPPD with solvents described by the SMD model. Terms in ALMO-EDA(sol) are represented with solid lines while the internal electrostatic interaction, denoted as "ELEC(0)", is shown in a dashed line.

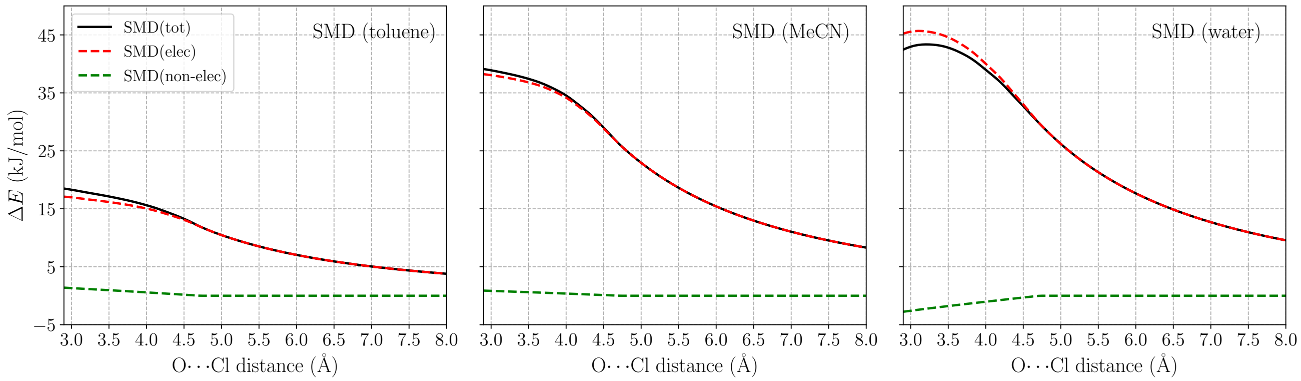

Figure S3: Electrostatic and non-electrostatic components (in kJ/mol) of the solvent contribution (ΔE_{SOL}) to the $H_2O\cdots Cl^-$ interaction with the $O\cdots Cl^-$ distance ranging from 2.9 to 8.0 Å, with solvents toluene, MeCN, and water modeled by SMD.

S2 Additional results for the [FeTPP($CO_2^{\bullet-}$)] derivatives

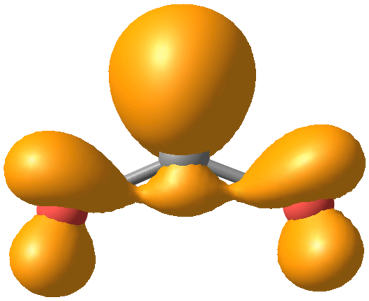

Figure S4: Spin density of $CO_2^{\bullet-}$ (isovalue: 0.08 a.u.) optimized with ωB97X-V/def2-TZVPP).

Table S2: Geometry distortion (GD) term for the CO_2 fragment with different fragmentation schemes (in kJ/mol).

Fragment	ΔE_{GD}
CO_2	206.6
$CO_2^{\cdot-}$	0.6

Table S3: Fe−CO_2 bond length (in Å) for all CO_2 adducts investigated in this study. All geometries are optimized with ωB97X-V/def2-TZVPP with PCM model for the CH_3CN solvent ($\epsilon = 35.88$).

Complex	r(Fe−C)
$[FeTPP(CO_2)]^{2-}$	2.08
[Fe-p-SUL-(CO_2)]$^{4-}$	2.09
[Fe-p-TMA-(CO_2)]^{0}	2.10
[Fe-o-TMA-(CO_2)]^{0}	2.06
[Fe-o-OH-(CO_2)]$^{2-}$	2.01
$[FeTPPF10(CO_2)]^{2-}$	2.10
[Fe-imid-(CO_2)]$^{-}$	2.07
[Fe-imid2-(CO_2)]^{0}	2.00

Table S4: ALMO-EDA(solv) results (in kJ/mol) with ωB97X-V/def2-TZVPP and PCM solvent (CH_3CN, $\epsilon = 35.88$) for all the different derivatives of the doubly reduced FeTPP$-CO_2$ adducts studied in this work.

Complex	ELEC	PAULI	DISP	POL	CT	INT
$[FeTPP(CO_2)]^{2-}$	-363.4	634.2	-66.2	-135.4	-123.0	-53.8
$[FeTPPF10(CO_2)]^{2-}$	-343.1	594.9	-66.0	-127.1	-106.0	-47.3
[Fe-*o*-OH-$(CO_2)]^{2-}$	-542.4	848.7	-88.5	-168.3	-167.5	-117.9
[Fe-*p*-OH-$(CO_2)]^{2-}$	-357.3	626.9	-66.1	-134.2	-111.7	-42.5
[Fe-*p*-TMA-$(CO_2)]^{0}$	-296.2	451.2	-71.7	-47.3	-111.0	-75.0
[Fe-*o*-TMA-$(CO_2)]^{0}$	-379.3	545.7	-96.2	-59.5	-129.7	-119.0
[Fe-*p*-SUL-$(CO_2)]^{4-}$	-343.1	613.9	-66.1	-132.6	-119.7	-47.6
[Fe-*o*-imid-$(CO_2)]^{-}$	-449.3	748.0	-100.0	-153.0	-149.2	-103.1
[Fe-*o*-imid2-$(CO_2)]^{0}$	-1005.4	955.3	-131.2	-201.4	-176.1	-197.3
[Fe-*o*-imid2-$(CO_2)]^{2-}$(NH-ref) [a]	-504.0	827.1	-91.3	-163.7	-155.5	-87.4

[a] The [Fe-*o*-imid2-$(CO_2)]^{2-}$(NH-ref) corresponds the reference calculation to separate the effect of the amino hydrogen bonds and the methylimidazolium. We replaced the methylimidazolium with methyl and kept the bond distances for both hydrogen bonds frozen.

Table S5: Gas phase ALMO-EDA results with ωB97X-V/def2-TZVPP (in kJ/mol) for the selected derivatives of the doubly reduced FeTPP$-CO_2$ complexes.

Complex	ELEC	PAULI	DISP	POL	CT	INT
$[FeTPP(CO_2)]^{2-}$	-135.5	600.6	-64.8	-182.2	-126.7	128.5
[Fe-*p*-SUL-$(CO_2)]^{4-}$	150.4	625.2	-65.8	-179.9	-121.8	408.1
[Fe-*o*-TMA-$(CO_2)]^{0}$	-401.0	444.6	-72.0	-95.1	-110.3	-233.8

S3 Additional results for the substituted terphenyl$\cdots CO_2$ complexes

Below we show the ALMO-EDA results for the reactant and product states of the substituted terphenyl$\cdots CO_2$ complex (carrying -1 charge) evaluated at the ωB97X-V/def2-TZVPD (Tables S6) and S7) and B3LYP-D3(BJ)/6-311G(d,p) levels of theory with or without solvent. Comparing the results in Table S7 and Table S9, we found that B3LYP-D3(BJ)/6-311G(d,p) produces much larger CT energies than ωB97X-V/def2-TZVPD for the product-state complexes when the solvent is absent. The differences in reactant state, on the other hand, are much more moderate. We ascribe this discrepancy to the more substantial delocalization error associated with the B3LYP functional,[1,2] which, as shown in our previous work,[3] can result in substantial overestimation of the CT component in ALMO-EDA (and correspondingly the total binding energy). Using the range-separated hybrid ωB97X-V functional, on the other hand, considerably reduces the spurious charge delocalization, and results in unconstrained SCF solutions for the reactant and product states in which the excess electron is well-localized on the terphenyl and CO_2 moieties, respectively. Table S10 shows the fragment Mulliken populations in the *fully relaxed* reactant and product states given by these two levels of theory. It reveals that the charge population on the CO_2 moiety largely deviates from -1 when calculated with B3LYP-D3(BJ) in vacuum, which, however, does not occur when ωB97X-V is employed. Interestingly, with the presence of SMD solvent, the ALMO-EDA results at these two different levels of theory become more comparable (see Tables S6 and S8), indicating that the solvent environment assists in stabilizing the charge-separated reactant and product states and mitigates the spurious charge delocalization associated with the B3LYP-D3(BJ)/6-311G(d,p) model chemistry. This further demonstrates the value of incorporating solvation effects in ALMO-EDA calculations for intermolecular complexes in solution, since otherwise the EDA results will suffer from artifacts caused by the unrealistic gas phase environment. The differences between the CT energies given by these two levels of theory now mainly arise from the larger basis set superposition error (BSSE) associated with the smaller 6-311G(d,p) basis set.

Table S6: ALMO-EDA(solv) results with **ωB97X-V/def2-TZVPD** (in kJ/mol) for the reactant- and product-state complexes of the electron-transfer reaction from terphenyl$^{\bullet-}$ to CO_2 **in CH_2Cl_2** ($\epsilon = 8.93$, described by the SMD model).

	Reactant state					
	ELEC	PAULI	DISP	POL	CT	INT
$-NMe_2$	-14.32	24.63	-21.27	-1.26	-2.81	-15.02
$-OH$	-12.61	21.31	-18.85	-1.06	-2.52	-13.73
$-CH_3$	-13.52	22.01	-17.99	-1.07	-2.58	-13.15
$-H$	-12.75	22.06	-19.29	-1.10	-2.62	-13.70
$-Br$	-11.92	22.27	-19.5	-1.10	-2.60	-12.86
$-CF_3$	-11.79	21.90	-19.56	-1.09	-2.50	-13.04
$-NO_2$	-10.81	21.12	-19.09	-1.03	-2.21	-12.02
	Product state					
	ELEC	PAULI	DISP	POL	CT	INT
$-NMe_2$	8.97	12.44	-16.80	-1.55	-1.10	1.96
$-OH$	5.79	13.28	-16.96	-1.56	-1.14	-0.58
$-CH_3$	5.97	13.44	-17.12	-1.60	-1.16	-0.46
$-H$	4.78	13.52	-17.04	-1.56	-1.15	-1.44
$-Br$	0.65	13.78	-17.07	-1.61	-1.17	-5.42
$-CF_3$	-1.26	14.02	-17.15	-1.60	-1.16	-7.15
$-NO_2$	-4.97	14.42	-17.19	-1.67	-1.20	-10.61

Table S7: ALMO-EDA results (in kJ/mol) with **ωB97X-V/def2-TZVPD** for the reactant- and product-state complexes of the electron-transfer reaction from terphenyl$^{\bullet-}$ to CO_2 **in the gas phase**.

	Reactant state					
	ELEC	PAULI	DISP	POL	CT	INT
$-NMe_2$	-19.05	26.29	-21.29	-3.58	-3.16	-20.80
$-OH$	-17.53	22.69	-18.81	-3.17	-2.81	-19.64
$-CH_3$	-17.71	23.75	-18.05	-3.31	-2.99	-18.31
$-H$	-17.35	23.41	-19.28	-3.35	-2.93	-19.50
$-Br$	-15.84	23.61	-19.52	-2.80	-2.85	-17.39
$-CF_3$	-15.32	22.92	-19.59	-2.69	-2.70	-17.38
$-NO_2$	-15.25	22.90	-19.39	-2.86	-2.57	-17.17
	Product state					
	ELEC	PAULI	DISP	POL	CT	INT
$-NMe_2$	35.10	15.16	-17.09	-24.87	-1.43	6.88
$-OH$	19.27	16.00	-17.25	-23.20	-1.53	-6.72
$-CH_3$	20.32	16.16	-17.43	-23.78	-1.56	-6.29
$-H$	15.58	16.26	-17.36	-22.84	-1.55	-9.92
$-Br$	-2.11	16.53	-17.42	-24.83	-1.63	-29.46
$-CF_3$	-12.37	16.80	-17.50	-24.06	-1.64	-38.77
$-NO_2$	-27.95	17.22	-17.57	-25.04	-1.73	-55.07

Table S8: ALMO-EDA(solv) results with **B3LYP-D3(BJ)/6-311G(d,p)** (in kJ/mol) for the reactant- and product-state complexes of the electron-transfer reaction from terphenyl$^{\bullet -}$ to CO_2 **in CH_2Cl_2** ($\epsilon = 8.93$, described by the SMD model). Note that counterpoise correction is *not* applied in these calculations since it is currently incompatible with the SMD model.

	Reactant state					
	ELEC	PAULI	DISP	POL	CT	INT
$-NMe_2$	-9.44	17.40	-19.38	-0.41	-8.54	-20.37
$-OH$	-8.47	15.08	-17.22	-0.34	-7.34	-18.29
$-CH_3$	-9.04	15.57	-16.60	-0.35	-7.43	-17.85
$-H$	-8.32	15.49	-17.45	-0.36	-7.53	-18.16
$-Br$	-7.67	15.98	-18.11	-0.37	-7.44	-17.61
$-CF_3$	-7.77	15.74	-17.95	-0.31	-7.36	-17.65
$-NO_2$	-7.05	15.40	-18.02	-0.27	-7.16	-17.10
	Product state					
	ELEC	PAULI	DISP	POL	CT	INT
$-NMe_2$	13.42	3.76	-9.72	-1.29	-11.04	-4.87
$-OH$	10.61	4.07	-9.90	-1.26	-11.69	-8.18
$-CH_3$	10.35	4.14	-9.99	-1.30	-11.95	-8.75
$-H$	9.30	4.16	-9.98	-1.26	-12.08	-9.85
$-Br$	5.62	4.19	-10.10	-1.31	-12.20	-13.81
$-CF_3$	3.86	4.19	-10.17	-1.30	-12.33	-15.75
$-NO_2$	0.24	4.32	-10.30	-1.38	-19.09	-26.21

Table S9: ALMO-EDA results (in kJ/mol) with **B3LYP-D3(BJ)/6-311G(d,p)** (counterpoise-corrected) for the reactant- and product-state complexes of the electron-transfer reaction from terphenyl$^{\bullet-}$ to CO_2 **in the gas phase**.

	Reactant state					
	ELEC	PAULI	DISP	POL	CT	INT
$-NMe_2$	-14.43	18.81	-19.40	-2.10	-2.87	-19.99
$-OH$	-13.88	16.31	-17.23	-1.96	-2.22	-18.99
$-CH_3$	-13.33	16.94	-16.63	-2.04	-2.83	-17.89
$-H$	-13.34	16.71	-17.48	-2.08	-2.42	-18.61
$-Br$	-11.92	17.19	-18.14	-1.68	-2.43	-16.98
$-CF_3$	-11.64	16.65	-17.98	-1.56	-2.29	-16.82
$-NO_2$	-10.19	16.30	-18.05	-1.22	-2.01	-15.16
	Product state					
	ELEC	PAULI	DISP	POL	CT	INT
$-NMe_2$	45.95	2.49	-9.72	-22.72	-8.98	7.02
$-OH$	30.65	2.68	-9.90	-20.77	-11.07	-8.42
$-CH_3$	29.77	2.73	-10.00	-21.43	-14.66	-13.59
$-H$	25.44	2.74	-9.98	-20.37	-15.60	-17.76
$-Br$	8.37	2.77	-10.11	-22.30	-23.77	-45.03
$-CF_3$	-2.01	2.75	-10.17	-21.38	-31.86	-62.67
$-NO_2$	-18.18	2.86	-10.30	-22.56	-74.96	-123.14

Table S10: Mulliken charge population (e^-) on the CO_2 moiety in the fully relaxed reactant and product states of the terphenyl$\cdots CO_2$ complex with different substituent groups. The calculations are performed at the B3LYP-D3(BJ)/6-311G(d,p) and ωB97X-V/def2-TZVPD levels of theory, without and with the SMD solvation model. The results show that gas-phase B3LYP-D3(BJ)/6-311G(d,p) calculations suffer from spurious charge transfer from $CO_2^{\bullet -}$ to the terphenyl moiety.

	B3LYP-D3(BJ) (vacuum)		ωB97X-V (vacuum)	
group	reactant	product	reactant	product
$-NMe_2$	-0.004	-0.753	0.010	-0.994
$-OH$	-0.004	-0.737	0.005	-0.989
$-H$	-0.004	-0.699	0.002	-0.989
$-CH_3$	-0.010	-0.706	0.008	-0.989
$-Br$	-0.001	-0.645	0.012	-0.985
$-CF_3$	0.000	-0.599	0.013	-0.986
$-NO_2$	0.004	-0.423	0.012	-0.981
	B3LYP-D3(BJ) (CH_2Cl_2)		ωB97X-V (CH_2Cl_2)	
group	reactant	product	reactant	product
$-NMe_2$	0.002	-0.977	0.014	-0.999
$-OH$	0.001	-0.974	0.009	-0.996
$-H$	0.001	-0.973	0.006	-0.995
$-CH_3$	-0.004	-0.974	0.013	-0.995
$-Br$	0.003	-0.972	0.013	-0.994
$-CF_3$	0.003	-0.972	0.015	-0.995
$-NO_2$	0.007	-0.716	0.020	-0.993

Table S11: Differential interaction energies (in kJ/mol) between the reactant and product complexes ($\Delta\Delta E_{\mathrm{INT}}$) and the differences between the monomer energies in the reactant and product complexes (ΔE_{PREP}, including contributions from both geometric distortion and change in electronic configuration). Ignoring the entropic contributions the free energy driving force for the electron transfer (ET) reaction can be approximated by $\Delta E_{\mathrm{ET}} = \Delta\Delta E_{\mathrm{INT}} + \Delta E_{\mathrm{PREP}}$.

	$\Delta\Delta E_{\mathrm{INT}}$	ΔE_{PREP}	ΔE_{ET}
$-NMe_2$	16.98	-50.45	-33.47
$-OH$	13.15	-32.45	-19.30
$-CH_3$	12.69	-20.82	-8.13
$-H$	12.26	-11.12	1.14
$-Br$	7.44	5.54	12.98
$-CF_3$	5.89	25.72	31.61
$-NO_2$	1.41	74.33	75.74

S4 Sample input for calculations using PCM

```
$molecule
    0 1
    --
    1 1
    Na 0.0  0.0  0.0
    --
    -1 1
    Cl 0.0  0.0  scan

    scan = 20.0
$end

$rem
    jobtype  eda
    eda2     2
    method   wb97x-v
    basis    def2-tzvppd
    scf_convergence  8
    thresh   14
    symmetry false
    sym_ignore true
    solvent_method pcm
$end

$pcm
    theory                      cpcm
    method                      swig
    solver                      inversion
    radii                       uff
    hpoints                     302
    heavypoints                 302
    vdwScale                    1.2
$end

$solvent
    dielectric                  80.0
$end
```

References

1 A. J. Cohen, P. Mori-Sánchez and W. Yang, *Science*, 2008, **321**, 792–794.

2 D. Hait and M. Head-Gordon, *J. Phys. Chem. Lett.*, 2018, **9**, 6280–6288.

3 Y. Mao, D. S. Levine, M. Loipersberger, P. R. Horn and M. Head-Gordon, *Phys. Chem. Chem. Phys.*, 2020, **22**, 12867–12885.
